# Effector *C*Las0185 targets methionine sulphoxide reductase B1 of *Citrus sinensis* to promote multiplication of ‘*Candidatus* Liberibacter asiaticus’ via enhancing enzymatic activity of ascorbate peroxidase 1

**DOI:** 10.1111/mpp.70002

**Published:** 2024-08-31

**Authors:** Shushe Zhang, Xuefeng Wang, Tingchang Zhao, Changyong Zhou

**Affiliations:** ^1^ Citrus Research Institute Southwest University, National Citrus Engineering Research Center Chongqing China; ^2^ State Key Laboratory for Biology of Plant Diseases and Insect Pests Chinese Academy of Agriculture Sciences, Institute of Plant Protection Beijing China

**Keywords:** ‘*Candidatus* Liberibacter asiaticus’, citrus huanglongbing, *C*Las0185, CsAPX1, CsMsrB1, plant immunity, reactive oxygen species

## Abstract

Citrus huanglongbing (HLB) has been causing enormous damage to the global citrus industry. As the main causal agent, ‘*Candidatus* Liberibacter asiaticus’ (*C*Las) delivers a set of effectors to modulate host responses, while the modes of action adopted remain largely unclear. Here, we demonstrated that CLIBASIA_00185 (*C*Las0185) could attenuate reactive oxygen species (ROS)‐mediated cell death in *Nicotiana benthamiana*. Transgenic expression of *C*Las0185 in *Citrus sinensis* ‘Wanjincheng’ enhanced plant susceptibility to *C*Las. We found that methionine sulphoxide reductase B1 (CsMsrB1) was targeted by the effector, and its abundance was elevated in *CLas0185*‐transgenic citrus plants. Their interaction promoted *C*Las proliferation. We then determined that CsMsrB1 sustained redox state and enzymatic activity of ascorbate peroxidase 1 (CsAPX1) under oxidative stress. The latter reduced H_2_O_2_ accumulation and was associated with host susceptibility to *C*Las infection. Consistently, citrus plants expressing *C*Las0185 and CsMsrB1 conferred enhanced APX activity and decreased H_2_O_2_ content. Taken together, these findings revealed how *C*Las0185 benefits *C*Las colonization by targeting CsMsrB1, which facilitated the antioxidant activity and depressed ROS during pathogen infection.

## Graphical abstract

Targeted by effector *C*Las0185, CsMsrB1 enhances enzymatic activity of CsAPX1 during ‘*Candidatus* Liberibacter asiaticus’ infection, which subtracts reactive oxygen species accumulation and compromises plant resistance.

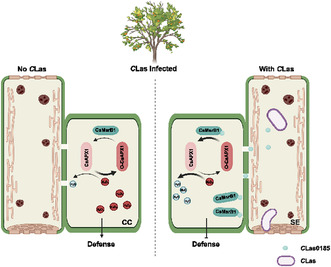

## 1 INTRODUCTION

Citrus huanglongbing (HLB) is the most devastating disease of citrus worldwide. The causal agents of HLB are three ‘*Candidatus* Liberibacter’ species, namely ‘*Ca*. Liberibacter asiaticus’ (*C*Las), ‘*Ca*. Liberibacter africanus’, and ‘*Ca*. Liberibacter americanus’, which are phloem‐limited and vectored by *Diaphorina citri* (Asian citrus psyllid, ACP) (Bóve, 2006). Among the three species, *C*Las is the most prevalent HLB pathogen. As no known commercial cultivars confer HLB resistance and none of the current strategies are efficient in preventing disease (Blaustein et al., 2018; Zhou, 2020), understanding the intricacy underlying HLB pathogenesis is necessary for the development of effective management strategies.

Secreted proteins of pathogens, called effectors, are prominent players to aid infection by manipulating plant immunity, which creates favourable environments for pathogen colonization and proliferation (Jones & Dangl, 2006). Genome sequence analysis of *C*Las revealed its ability to deliver effectors into host cells using a general Sec‐dependent secretion apparatus (Duan et al., 2009). Prasad et al. (2016) characterized 86 Sec‐dependent *C*Las effectors, and many of them have been identified according to their functions on plant immune responses. For instance, *C*Las4425 induces cell death in *Nicotiana benthamiana* and enhances citrus susceptibility to *C*Las by interfering with salicylic acid‐mediated plant immunity (Zhang, Wang, et al., 2023). Phylogenetic analysis indicated that effectors are highly conserved between *C*Las isolates but not across all three HLB species (Thapa et al., 2020).

The inability of the HLB pathogens to be cultured in vitro and the time‐consuming generation of transgenic citrus plants restrict the dissection of HLB pathogenicity. The molecular mechanisms of a few *C*Las effectors in *Citrus–C*Las interactions have been discovered: SDE1 (*C*Las Sec‐delivered effector 1) inhibits immune protease activity to suppress citrus defence (Clark et al., 2018). SDE15 suppression of plant immunity is dependent on accelerated cell death 2 (ACD2) (Pang et al., 2020). SDE3 and SDE4405 promote pathogen infection via manipulating host autophagy (Shi, Gong, et al., 2023; Shi, Yang, et al., 2023). A prophage‐encoded effector, AGH17488, targets ascorbate peroxidase 6 (CsAPX6) to scavenge reactive oxygen species (ROS) accumulation (Du et al., 2023). However, it is far from sufficient to fully elucidate how *C*Las effectors contribute to HLB development.

In plants, physiological responses to environmental constraints disrupt internal balance, among which redox homeostasis is largely manipulated during plant–pathogen interactions (Bleau & Spoel, 2021; Camejo et al., 2016). ROS are a group of molecules derived from O_2_, consisting of hydrogen peroxide (H_2_O_2_), hydroxyl radical (OH^−^), and singlet oxygen (^1^O_2_), which serve as a crucial component in redox homeostasis, and contribute to plant immunity (Mittler, 2017). Maintaining a basal level of ROS is necessary for life, while excessive ROS are toxic and cause oxidative stress. Thereby plants confer a complex array of systems to neutralize the effect, such as antioxidant system (Caverzan et al., 2012). This system comprises enzymatic antioxidants and some low molecular mass antioxidants. Among the enzymes, APX family proteins have a high affinity for H_2_O_2_ and contribute greatly to H_2_O_2_ detoxification (Sofo et al., 2015). They are involved in physiological responses, such as leaf senescence, and programmed cell death (de Pinto et al., 2013; Ribeiro et al., 2017). Besides the antioxidant system, the repair system is also associated with redox homeostasis, in which methionine sulphoxide reductase (Msr) family proteins are the main player (Rey & Tarrago, 2018).

*C*Las0185, delivered via the Sec translocon, comprises an N‐terminal signal peptide with 19 amino acids (aa) and the mature form with 49 aa (Prasad et al., 2016). Due to its high level of amino acid sequence identity among *C*Las strains, *C*Las0185 was predicted to be a core effector (Thapa et al., 2020). Using transient expression and genetic approaches, we identified that *C*Las0185 manipulates plant defence responses and contributes to HLB progression. Screening for the targets in citrus plants, we confirmed the interaction between *C*Las0185 and CsMsrB1. Using *Agrobacterium rhizogenes*‐mediated hairy root transformation in a *CLas0185*‐transgenic *Citrus sinensis* background, we determined that *C*Las0185 contribution to HLB pathogenicity is dependent on CsMsrB1. We further verified the association between CsMsrB1 and CsAPX1, and the latter was susceptible for HLB. Here, we conducted a time‐saving approach to analyse the role of effector‐interacting protein in HLB development with hairy root transformation. Moreover, the study provided a detailed examination of the molecular events associated with the depression of plant defence responses in *C*Las‐accumulated tissues.

## 2 RESULTS

### 2.1 *C*Las0185 suppresses ROS‐mediated cell death in *N*. *benthamiana*

**FIGURE 1:**
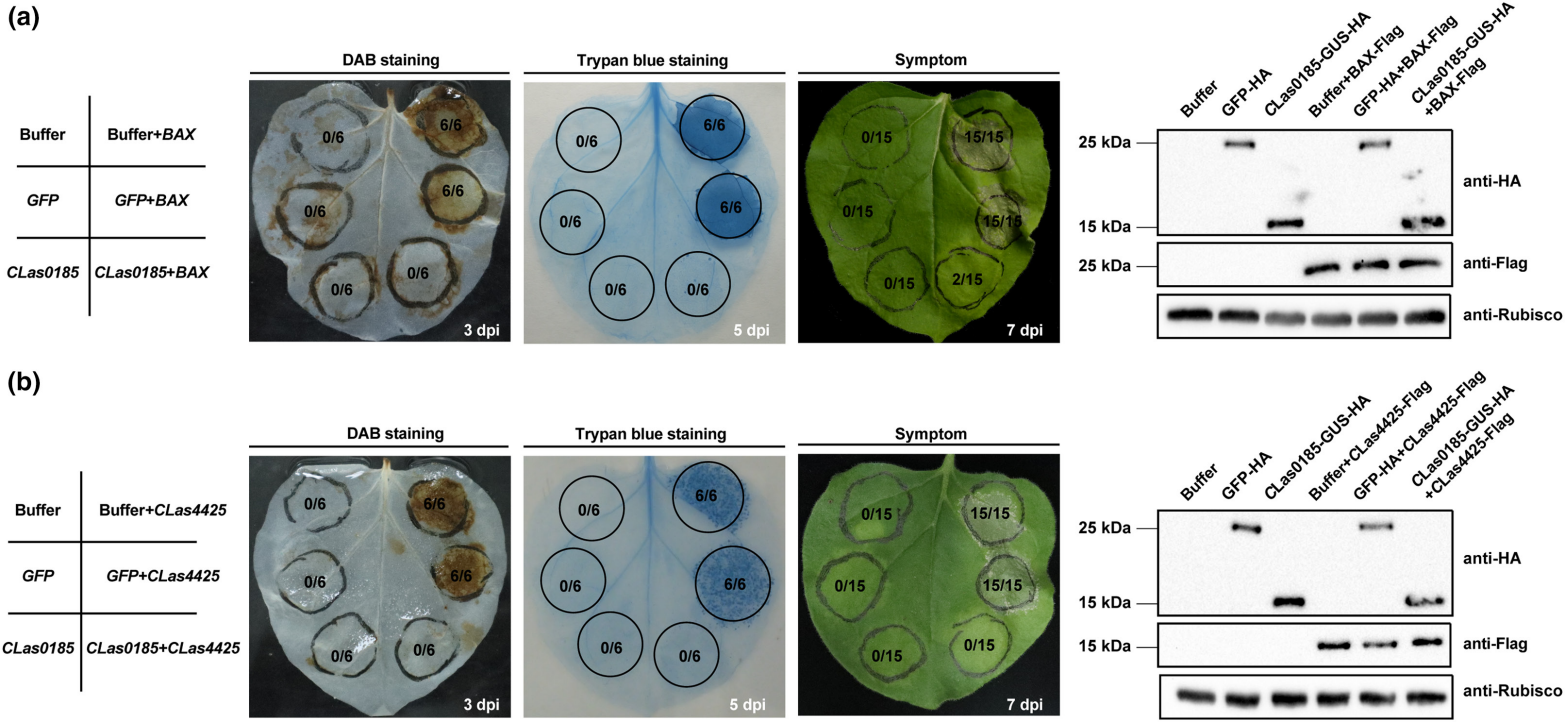
CLIBASIA_00185 (*C*Las0185) attenuates hypersensitive cell death in *Nicotiana benthamiana*. *C*Las0185 blocks (a) pro‐apoptotic mouse protein BCL2‐associated X protein (BAX)‐induced cell death and (b) *C*Las4425‐induced cell death. Leaves were infiltrated with buffer or *Agrobacterium tumefaciens* cells expressing *C*Las0185 or green fluorescent protein (GFP), either alone or followed 24 h later with *A*. *tumefaciens* cells harbouring BAX or *C*Las4425. The circles indicate infiltration sites. To visualize the reactive oxygen species burst, leaves were detached at 3 days post‐inoculation (dpi) for 3,3′‐diaminobenzidine (DAB) staining. The leaves for programmed cell death observation were photographed at 5 dpi after trypan blue staining. For symptom observation, leaves were collected and photographed at 7 dpi. Anti‐HA and anti‐FLAG antibodies were used to detect the expression of the indicated constructs at 3 dpi, and equal loading of each sample was confirmed by immunoblotting of RuBisCO. All experiments were performed in triplicate. The numbers in each circled area indicate summary data representing the number of infiltrated area(s) exhibiting hypersensitive responses over the total number of leaf areas infiltrated with a particular construct or combination of constructs.

Based on the Sec‐dependent *C*Las effectors identified by Prasad et al. (2016), we employed a functional screen using potato virus X (PVX) vector pGR107 to select potential virulence factors. In this assay, pro‐apoptotic mouse protein BCL2‐associated X protein (BAX) and green fluorescent protein (GFP) served as the positive and negative controls, respectively. The results indicated that *C*Las0185 attenuates the BAX‐induced ROS burst and cell death (Figure 1a). Intriguingly, it could also block the cell death‐inducing activity of *C*Las04425 (Figure 1b), which was previously determined to trigger cell death in *N*. *benthamiana* (Zhang, Wang, et al., 2023). Immunoblot verified that all the recombinant proteins were properly expressed at the expected size (Figure 1).

### 2.2 *C*Las0185 enhances plant susceptibility to *C*Las

**FIGURE 2:**
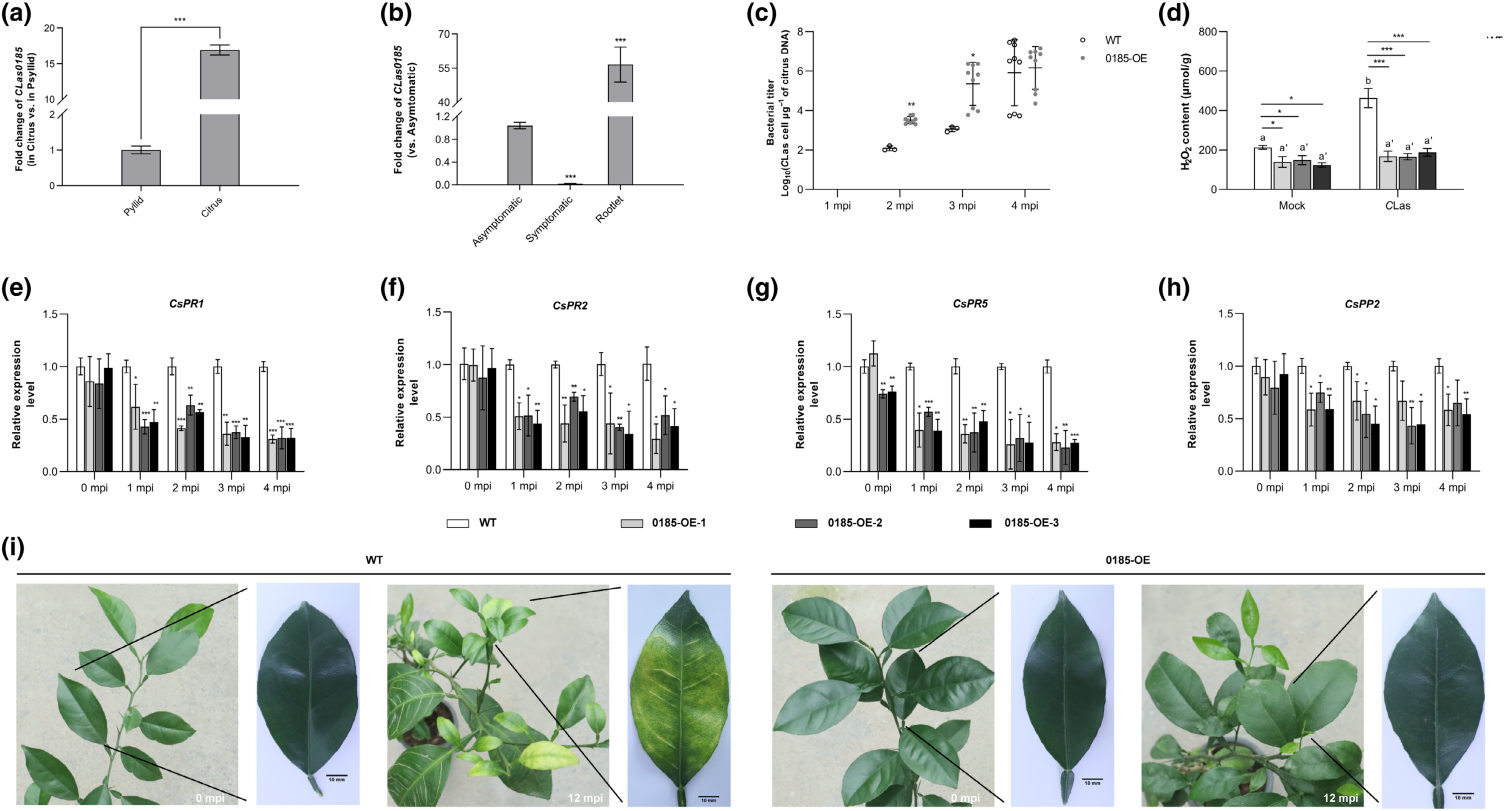
*C*Las0185 impairs immune responses in *Citrus sinensis*. (a, b) Comparison of *CLas0185* expression between psyllids and citrus (a), within a/symptomatic citrus leaves and rootlets (b). RNA was extracted from psyllid colonies and citrus plants that were infected by ‘*Candidatus* Liberibacter asiaticus’ (*C*Las), and tissue samples were collected from the same citrus plants. Transcript levels of *CLas0185* were determined by reverse transcription‐quantitative PCR (RT‐qPCR) with *CLasgryA* (GenBank no. CP001677.5) as the internal reference. (c) Quantitative analysis of *C*Las growth during a 4‐month period after graft inoculation in CLas0185‐transgenic citrus lines (0185‐OE) and wild‐type (WT) group. *C*. *sinensis* ‘Wanjincheng’ was used for the generation of transgenic citrus. WT Wanjincheng plants of the same age were used as controls. mpi = months post‐inoculation. The bacterial populations (*C*Las cells per μg of citrus DNA) were determined using quantitative PCR. (d) H_2_O_2_ accumulation in leaves of 0185‐OEs and WTs. The differences were determined by Student's *t* test, and different letters indicate statistically differences (*p* < 0.05). (e–h) Relative expression levels of pathogenesis‐associated genes (*PR*s) and phloem protein 2 gene (*PP2*) in citrus plants. Transcript levels measured with RT‐qPCR were normalized to levels in WTs using the *CsGAPDH* as endogenous control. Data in (a–h) represent mean ± *SD*. Asterisks indicate significant differences determined by Student's *t* test (*p < 0.05; **p < 0.01; ***p < 0.001; *n* = 3). The experiments comprised three independent biological replicates, and three technical repeats were performed. (i) *C*Las0185 attenuates huanglongbing symptom development. Images were taken at 0 and 12 months post‐inoculation. Scale bar: 10 mm.

The expression profiles of *CLas0185* were analysed to investigate its role in *C*Las virulence. The results showed that *CLas0185* was highly expressed (c. 17‐fold) in infected citrus plants compared to that in psyllids (Figure 2a). Within different citrus tissues, it was largely expressed in rootlets (Figure 2b). According to the importance of roots in *C*Las early infection (Johnson et al., 2014), our data imply that *C*Las0185 could have an effect on the initiation of pathogen colonization.

To determine the contribution of *C*Las0185 to disease progression, *CLas0185*‐transgenic citrus plants (0185‐OE) were generated via *Agrobacterium*‐mediated transformation, which was confirmed with PCR and β‐glucuronidase (GUS) assays (Figure S1a–c). Three independent 0185‐OE lines were obtained, and the transcript levels of *CLas0185* were detected with reverse transcription‐quantitative PCR (RT‐qPCR) (Figure S1d). They showed no difference in growth phenotype compared with wild‐type (WT) plants (Figure S1e,f). Citrus plants were graft‐inoculated using stems from diseased trees. A significant increase of *C*Las titres was detected in the 0185‐OE group compared to the WT group at 2 and 3 months post‐inoculation (mpi) (Figure 2c). Hence, *C*Las0185 accelerated pathogen multiplication during the early stages of infection.

As elicitor‐triggered ROS burst was inhibited in *N*. *benthamiana* expressing *CLas0185*, we examined H_2_O_2_ accumulation in leaves of 0185‐OEs. Before infection, H_2_O_2_ content was depressed in 0185‐OEs, indicating that the effector compromised citrus basal immunity (Figure 2d). At 6 mpi, H_2_O_2_ levels were elevated in the WT group compared to 0185‐OEs. We further monitored the transcript levels of plant defence‐related genes in citrus leaves, including pathogenesis‐related genes (*CsPR*s) and phloem protein 2 (*CsPP2*) within 4 mpi. We found their expression was markedly depressed in 0185‐OEs at 1 mpi compared with that in the WT group (Figure 2e–h). Of interest, HLB symptoms observed at 12 mpi were attenuated in 0185‐OEs (Figure 2i). Taken together, our findings demonstrate that *C*Las0185 enhances citrus susceptibility to *C*Las.

### 2.3 *C*Las0185 contribution to HLB pathogenicity is dependent on CsMsrB1

To understand the potential role of *C*Las0185 in citrus, we performed a yeast two‐hybrid (Y2H) assay using a *C*. *sinensis* cDNA library to identify candidate *C*Las0185‐interacting proteins (Table S1). Among them, the *C*. *sinensis* protein annotated as CsMsrB1 (NCBI accession XP_006477977.2) is implicated in redox homeostasis (Rey & Tarrago, 2018). It has been reported that Msr proteins play roles in plant response to biotic stresses (Fu et al., 2023; Gao et al., 2012); however, little is known about the underlying mechanisms. Hence, we chose CsMsrB1 for further study. As MsrB1 belongs to the Msr family, a phylogenetic tree of Msr proteins was constructed from *Nicotiana* *tabacum* (10), *Zea mays* (3), *Musa acuminata* (6), and *C*. *sinensis* (6). Msr proteins in citrus are divided into two subfamilies; CsMsrB1 is in Cluster VI belonging to MsrB subgroup (Figure S2). The MsrB subfamily proteins possess a conserved SelR domain, which plays a role in maintaining intracellular redox homeostasis via reducing methionine sulphoxide to methionine (Figure S2).

**FIGURE 3:**
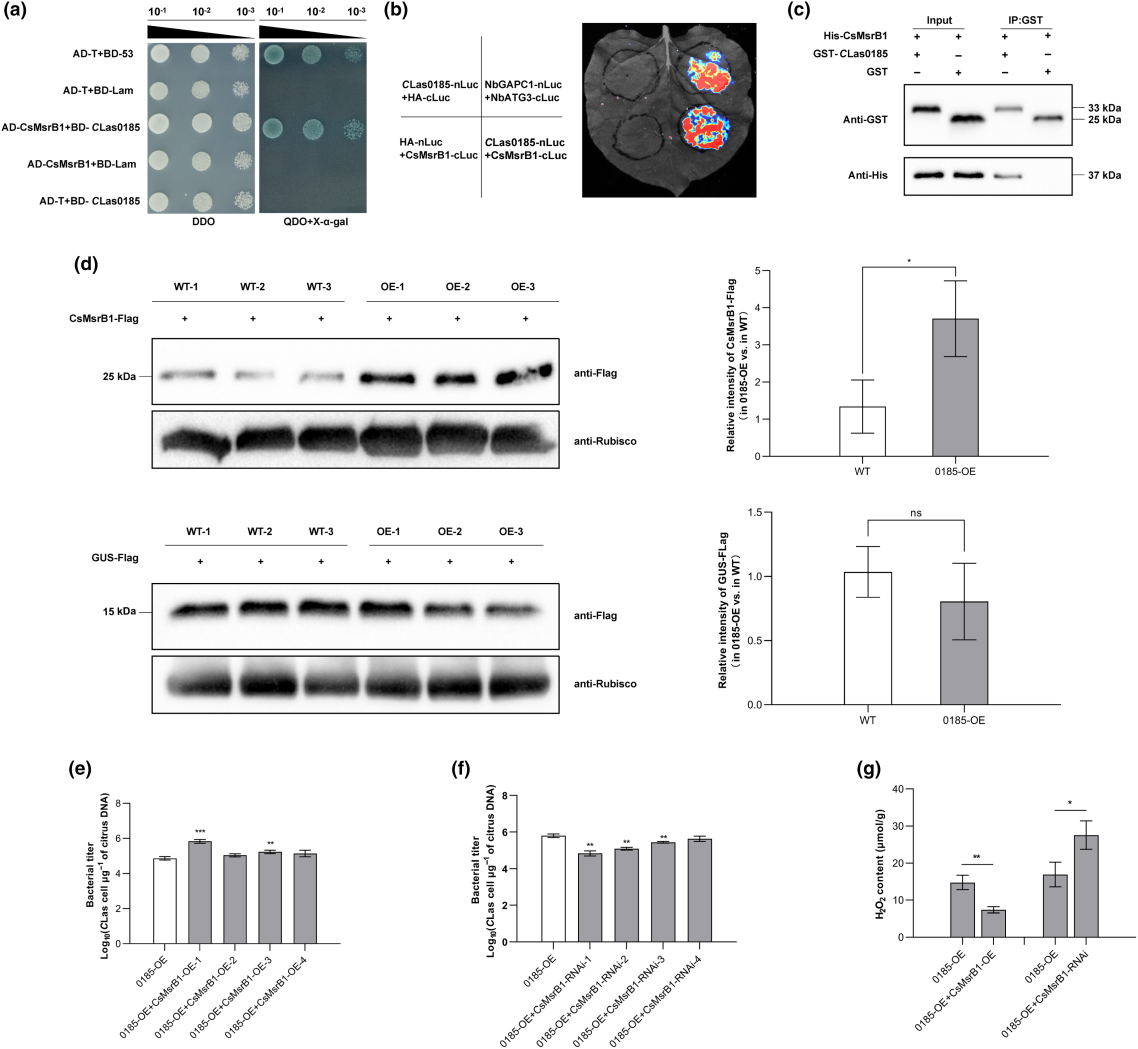
The interaction between *C*Las0185 and CsMsrB1 elevates CsMsrB1 abundance in *Citrus sinensis* and promotes host susceptibility to ‘*Candidatus* Liberibacter asiaticus’ (*C*Las). (a) *C*Las0185 binds with CsMsrB1 in a yeast two‐hybrid (Y2H) assay. Yeast co‐transformed with AD‐T + BD‐53 and AD‐T + BD‐Lam served as the positive and negative controls, respectively. Yeast co‐transformed with AD‐CsMsrB1 + BD‐Lam and AD‐T + BD‐*C*Las0185 were auto‐activation controls. Serial 10‐fold dilutions of co‐transformed yeast cells on double‐dropout (DDO) and quadruple‐dropout (QDO) + X‐α‐gal are shown. (b) Luciferase complementation imaging assay confirmed the interaction between *C*Las0185 and CsMsrB1 in the leaves of *Nicotiana benthamiana* at 3 days post‐infiltration (dpi). The N‐ or C‐terminal fragments of luciferase were fused to the C‐terminus of *C*Las0185 and CsMsrB1. Co‐expression of NbGAPC1‐nLuc + NbATG3‐cLuc was used as a positive control, and co‐expression of *C*Las0185‐nLuc + HA‐cLuc or HA‐nLuc + CsMsrB1‐cLuc was applied as negative controls. (c) Glutathione‐S‐transferase (GST) pull‐down assay demonstrated the interaction between *C*Las0185 and CsMsrB1. GST‐*C*Las0185 and GST were expressed in *Escherichia coli*, immobilized on glutathione sepharose beads, and incubated with *E*. *coli* lysate containing His‐CsMsrB1. Input and eluted (IP) proteins were immunoblotted using the anti‐His and anti‐GST antibodies. (d) *C*Las0185 increased the abundance of CsMsrB1. CsMsrB1 and GUS fused with FLAG were expressed in 0185‐OE and wild‐type (WT) groups through agro‐infiltration. Proteins were extracted at 3 dpi, and the abundances of CsMsrB1 and β‐glucuronidase (GUS) in 0185‐OEs compared to those in WTs were determined with an anti‐FLAG antibody. RuBisCO served as the endogenous control. Band intensity in blots was calculated by ImageJ based on three biological replicates, and the relative intensity was calculated. The experiment contained three independent biological replicates. (e, f) *C*Las titres in *CsMsrB1*‐overexpressing (CsMsrB1‐OE) and ‐silenced (CsMsrB1‐RNAi) 0185‐OEs. *Agrobacterium rhizogenes*‐mediated hairy root genetic transformation was conducted to manipulate *CsMsrB1* expression in *C*Las‐infected 0185‐OEs. Bacterial growth was detected at 3 months post‐inoculation using TaqMan quantitative PCR. The experiment contained four independent biological replicates, and three technical repeats were performed. (g) Co‐expression of *C*Las0185 and CsMsrB1 reduces H_2_O_2_ accumulation. The H_2_O_2_ content was measured in hairy roots of CsMsrB1‐OEs and CsMsrB1‐RNAis in a 0185‐OE background. 0185‐OE served as the control. Data in (d–g) represent mean ± *SD*. Asterisks indicate significant differences determined by Student's *t* test (*p < 0.05; **p < 0.01; ***p < 0.001; ns, no significance). The experiments were performed twice, with similar results.

To validate the interaction of *C*Las0185 and CsMsrB1, a pairwise Y2H assay was performed. All yeast transformants grew on control plates. Nevertheless, only the transformants containing *C*Las0185 and CsMsrB1 could induce β‐galactosidase activity and exhibited a positive signal on stringent SD/−Leu/−Trp/−His/−Ade medium (Figure 3a). We next conducted a luciferase complementation imaging (LCI) assay in *N*. *benthamiana* to validate the interaction in vivo. In this assay, luciferase complementation indicated that *C*Las0185 bound with CsMsrB1, observed with chemiluminescence apparatus at 3 dpi (Figure 3b). We further used a pull‐down assay and confirmed the interaction between *C*Las0185 and CsMsrB1 in vitro (Figure 3c).

We then analysed the abundance of CsMsrB1 to see if it could be affected by the effector. *CsMsrB1‐FLAG* and *GUS‐FLAG* were transiently expressed in leaves of either 0185‐OE or WT group, and total proteins were extracted at 3 dpi. The abundance of CsMsrB1‐FLAG was notably higher in transgenic plants than in WT lines, while no significant difference was observed between 0185‐OEs and WTs in the expression of GUS‐FLAG (Figure 3d). The results indicated that *C*Las0185 increased the abundance of CsMsrB1 in *C*. *sinensis*.

To explore whether CsMsrB1 is involved in the *C*Las0185‐mediated promotion of *C*Las infection, we generated *CsMsrB1*‐overexpressing (CsMsrB1‐OE) and *CsMsrB1*‐silenced (CsMsrB1‐RNAi) transgenic citrus hairy roots using *Agrobacterium rhizogenes*‐mediated transformation, which developed from *C*Las‐infected 0185‐OE stem sections. The derived rootlets were verified with PCR at 3 mpi. Four CsMsrB1‐OE and CsMsrB1‐RNAi lines were obtained, and the overexpression/silencing efficiency was determined using RT‐qPCR (Figure S3). The control groups were the rootlets derived from *C*Las‐infected 0185‐OE stem sections without transformation. In 0185‐OE + CsMsrB1‐OEs, *C*Las titres were increased while H_2_O_2_ content was reduced; in 0185‐OE + CsMsrB1‐RNAi lines, *C*Las titres were decreased while H_2_O_2_ content was elevated (Figure 3e–g). The results indicated that CsMsrB1 contributes to *C*Las0185‐facilitated HLB development via inhibiting ROS accumulation.

### 2.4 CsMsrB1 regulates redox state and activity of CsAPX1

**FIGURE 4:**
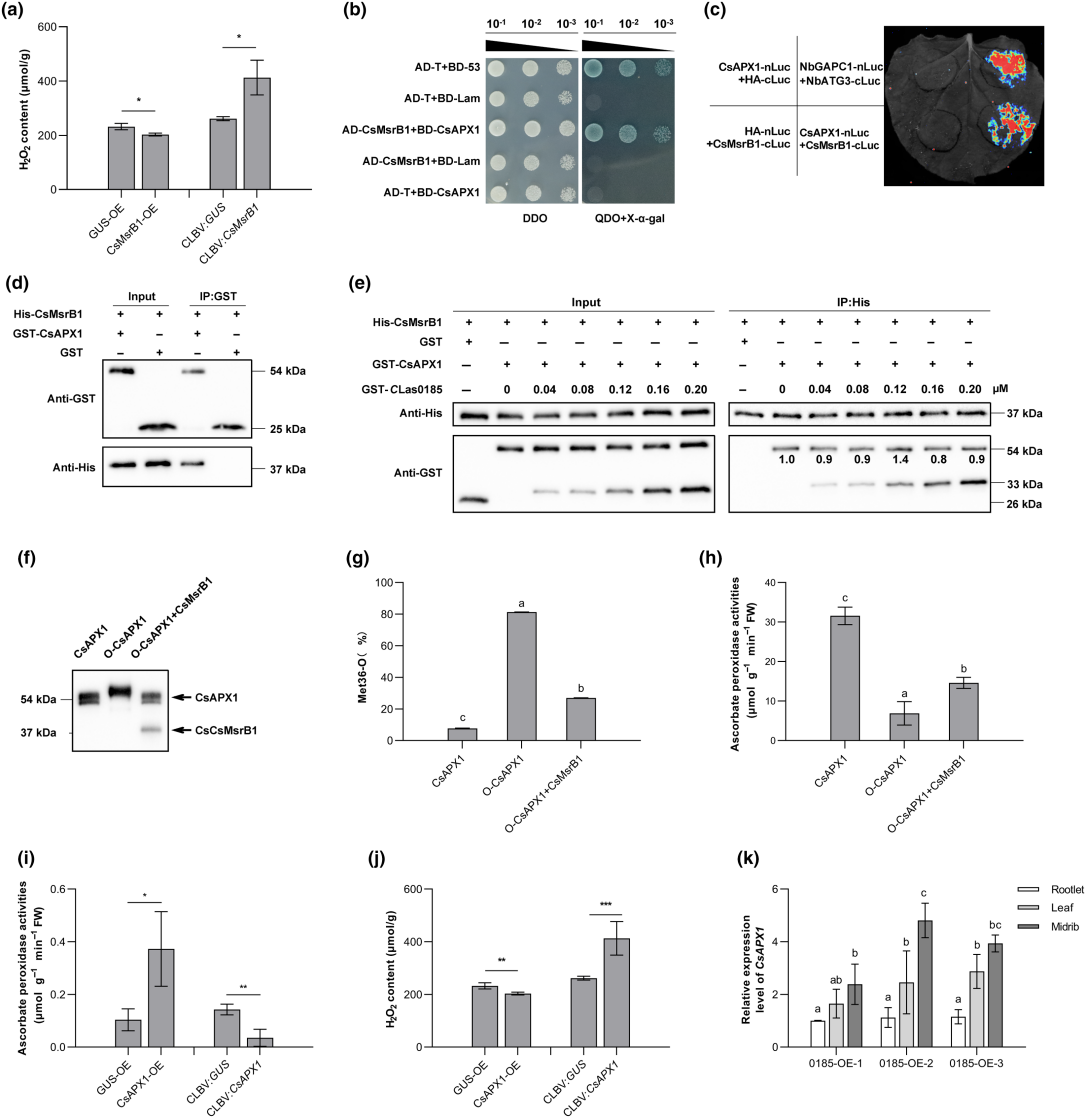
CsMsrB1 regulates the redox state and activity of CsAPX1. (a) CsMsrB1 impairs H_2_O_2_ accumulation in citrus plants. *CsMsrB1*‐overexpressing (CsMsrB1‐OE) and ‐silenced (CLBV:*CsMsrB1*) *Citrus sinensis* ‘Wanjincheng’ were generated. GUS‐OE and CLBV:*GUS* served as controls, respectively. (b–d) The interaction between CsMsrB1 and CsAPX1 was validated using yeast two‐hybrid assay, luciferase complementation imaging, and pull‐down assay. (e) *C*Las0185 did not compete with CsAPX1 for binding to CsMsrB1. Protein compounds containing 0.2 μM CsMsrB1, CsAPX1, as well as *C*Las0185 with corresponding dilutions were prepared and immobilized on Ni sepharose beads. Input and eluted (IP) proteins, namely His‐CsMsrB1, GST, GST‐*C*Las0185, and GST‐CsAPX1, were immunoblotted using anti‐His and anti‐GST antibodies. Band intensity in blots was calculated by ImageJ. (f) Oxidized CsAPX1 (O‐CsAPX1) was reduced by CsMsrB1. Oxidized by H_2_O_2_, O‐CsAPX1 has a higher molecular weight than CsAPX1. (g) The percentage change of Met36‐O in the native, oxidized and reduced CsAPX1. Met36 was detected by liquid chromatography–tandem mass spectrometry. The percentage changes of Met36‐O are 7.73% in the native CsAPX1, 81.33% in O‐CsAPX1, and 27% in CsMsrB1‐reduced O‐CsAPX1. (h) CsMsrB1 restored the ascorbate peroxidase activity of O‐CsAPX1. (i, j) CsAPX1 enhanced ascorbate peroxidase activity and decreased H_2_O_2_ accumulation in *C*. *sinensis*. *CsAPX1*‐overexpressing (CsAPX1‐OE) and ‐silenced (CLBV:*CsAPX1*) Wanjincheng plants were generated. GUS‐OE and CLBV:*GUS* served as controls, respectively. Data in (a, i, and j) represent mean ± *SD*. Asterisks indicate significant differences determined by Student's *t* test (*p < 0.05; **p < 0.01; ***p < 0.001; *n* = 3). (k) *CsAPX1* expression analysis in rootlets, leaves and midribs of 0185‐OE. Data in (g, h, k) represent mean ± *SD*. Different lowercase letters indicate significant differences as measured with Fisher's LSD test (*p* = 0.05, *n* = 3). The experiments were performed twice, with similar results.

Because CsMsrB1 is a prominent player in redox homeostasis, we determined H_2_O_2_ content in leaves of *CsMsrB1*‐overexpressing/silenced citrus plants. Its expression was manipulated through homologous transient overexpression and citrus leaf blotch virus (CLBV)‐mediated virus‐induced gene silencing (VIGS) assay in *C*. *sinensis*. *CsMsrB1* overexpression was validated by immunoblotting at 3 dpi, and the fragment insertion was determined with RT‐PCR at 1 mpi (Figure S4a,b). The transcript levels of *CsMsrB1* were examined with RT‐qPCR (Figure S4c,d). The overexpression of *CsMsrB1* resulted in a significant decrease in basal H_2_O_2_ levels in citrus leaves, while its silencing enhanced H_2_O_2_ accumulation (Figure 4a). Our findings provide direct evidence that CsMsrB1 is involved in scavenging H_2_O_2_.

Previous studies verified the interplay between Msr proteins and enzymatic antioxidants (Cui et al., 2022; Xiao et al., 2021). Hence, we examined the interaction between CsMsrB1 and six enzymatic antioxidants, namely glutathione reductase (GR), monodehydroascorbate reductase (MDAR), superoxide dismutase (SOD) [Fe], SOD[Cu‐Zn], ascorbate peroxidase 1 (APX1), and catalase 1 (CAT1). Using a Y2H assay, we confirmed physical interactions of CsMsrB1‐CsAPX1 and CsMsrB1‐CsCAT1 (Figures 4b and S5). Because CsAPX6 was targeted by another *C*Las effector, AGH17488 (Du et al., 2023), we focused on the interaction between CsMsrB1 and CsAPX1, which was further validated with LCI and pull‐down assays (Figure 4c,d). A phylogenetic tree of APXs was constructed from *C*. *sinensis* (6), *N*. *tabacum* (3), *Arabidopsis* (6), and *Z*. *mays* (7), indicating a high degree of amino acid homology between CsAPX1 and AtAPX1 (Figure S6). Alignment of amino acid sequences of CsAPXs was analysed with the percentage sequence identities from 11.24% to 77.35% (Figure S6). CsMsrB1 did not interact with CsAPXs in a pairwise Y2H assay apart from with CsAPX1 (Figure S6). Using a competitive pull‐down assay, we clarified that the binding between CsMsrB1 and CsAPX1 was not weakened by *C*Las0185 (Figure 4e).

Recombinant proteins were prepared to determine the effect of CsMsrB1 on CsAPX1 activity. CsAPX1 oxidized by H_2_O_2_ has a higher molecular weight than the unoxidized (native) form. Added with CsMsrB1, oxidized CsAPX1 (O‐CsAPX1) could be reduced, and the band in SDS‐PAGE shifted back to the position of the unoxidised CsAPX1 (Figure 4f). Liquid chromatography–tandem mass spectrometry (LC–MS/MS) analysis indicated that the percentage of Met36‐O increased significantly in O‐CsAPX1, while it decreased in substrates added with CsMsrB1 (Figure 4g). Enzymatic analysis revealed that oxidization hindered CsAPX1 activity, which could be partially restored by CsMsrB1 (Figure 4h).

Homologous overexpression and VIGS assays were conducted to manipulate *CsAPX1* expression in *C. sinensis*. After verification of protein expression at 3 dpi and the fragment insertion at 1 mpi, the overexpressing/silencing efficiency was determined using RT‐qPCR (Figure S7). We then examined APX activity and H_2_O_2_ in citrus plants. Overexpression of *CsAPX1* significantly elevated APX activity and reduced the H_2_O_2_ level in planta, whereas silencing it decreased APX activity and increased H_2_O_2_ accumulation (Figure 4h,i). The *CsAPX1* expression in 2‐year‐old 0185‐OEs was determined with RT‐qPCR, indicating that it was expressed in rootlets, leaves, and midribs. *CsAPX1* expression in rootlets was lower than in leaves and midribs (Figure 4k). From our findings, we speculate that CsMsrB1 eliminates ROS by sustaining CsAPX1 activity.

### 2.5 *CsAPX1* is a susceptibility gene for HLB

**FIGURE 5:**
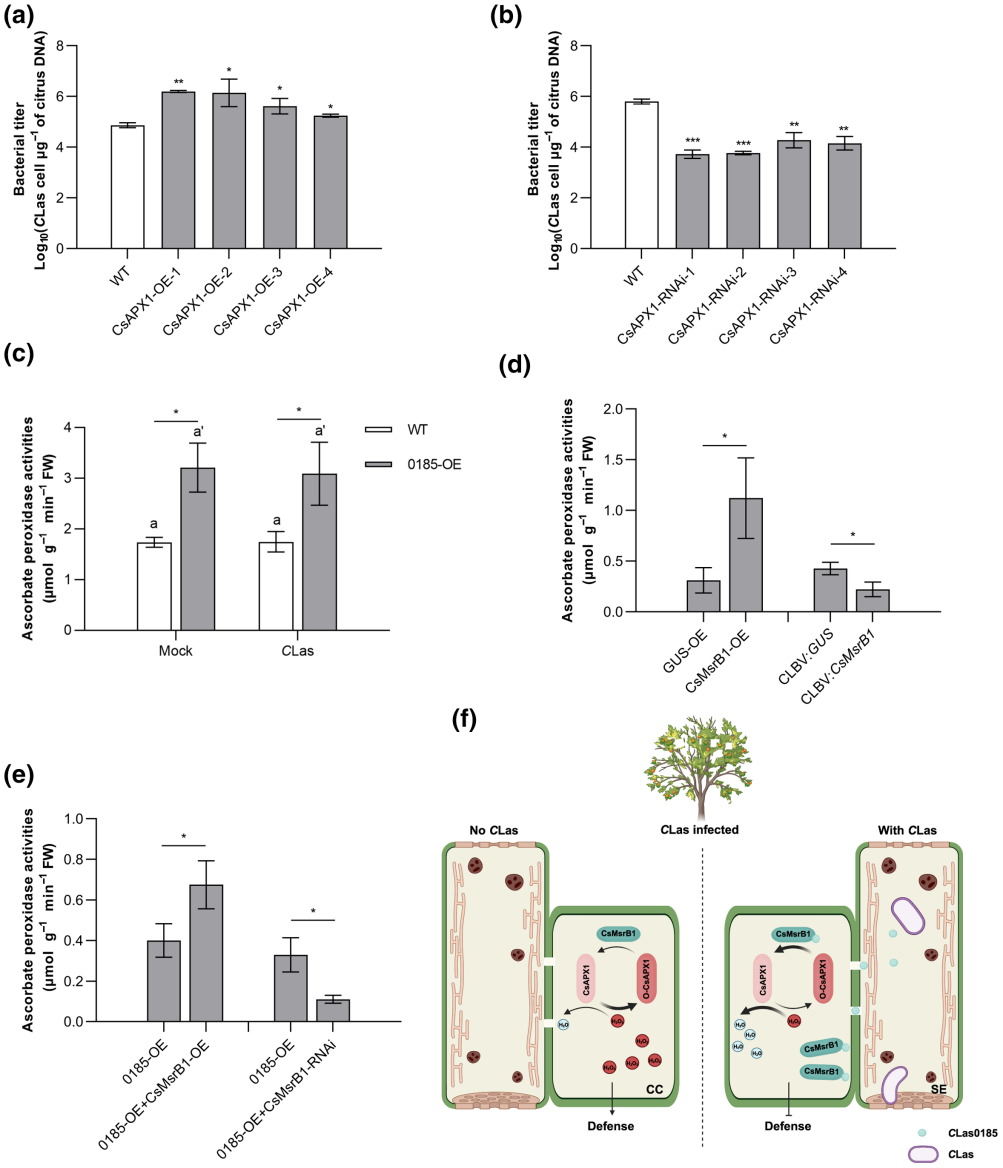
CsAPX1 promotes ‘*Candidatus* Liberibacter asiaticus’ (*C*Las) proliferation. (a, b) *C*Las titres in CsAPX1‐OE/RNAi lines. *Agrobacterium rhizogenes*‐mediated hairy root genetic transformation was conducted to manipulate *CsAPX1* expression in huanglongbing‐diseased citrus plants. Bacterial growth was detected at 3 months post‐inoculation (mpi) using TaqMan quantitative PCR. The experiment contained four independent biological replicates, and three technical repeats were performed. (c–e) Detection of ascorbate peroxidase (APX) activity. APX activity was detected in citrus plants expressing *CLas0185* and *CsMsrB1*, as well as in rootlets co‐expressing *CLas0185* and *CsMsrB1*. Wild type (WT) served as the control for 0185‐OE. GUS‐OE and CLBV:*GUS* were used as controls for CsMsrB1‐OE and CLBV:*CsMsrB1*, respectively. 0185‐OE served as the control for 0185‐OE + CsMsrB1‐OE and 0185‐OE + CsMsrB1‐RNAi, respectively. In (c), the differences were determined by Student's *t* test, and letters represent the difference between Mock and *C*Las inoculation (*p* < 0.05; *n* = 3). Data in (a–e) represent mean ± *SD*. Asterisks indicate significant differences determined by Student's *t* test (*p < 0.05; **p < 0.01; ***p < 0.001; *n* = 3). The experiments were performed twice, with similar results. (f) A schematic diagram illustrating that *C*Las0185 depresses plant immunity through targeting CsMsrB1. Perceived by hosts, *C*Las induces plant defence responses, including the reactive oxygen species (ROS) burst. To suppress plant immunity, *C*Las effector *C*Las0185 is delivered, which targets CsMsrB1 and increases its abundance. The latter restores the activity of CsAPX1, which scavenges H_2_O_2_ accumulation, which negatively regulates plant defence. CC, companion cell; SE, sieve element.

To determine the role of *CsAPX1* in *C*Las infection, we generated CsAPX1‐OE and CsAPX1‐RNAi transgenic citrus hairy roots, which were developed from *C*Las‐infected stem sections. Using PCR and GUS staining at 3 mpi, four CsAPX1‐OE and CsAPX1‐RNAi lines were confirmed, and the overexpressing/silencing efficiency was determined using RT‐qPCR (Figure S8). Rootlets derived from *C*Las‐infected stem sections without transformation served as control groups. Determined with TaqMan qPCR, *C*Las titres were increased in the CsAPX1‐OE group, while decreased in the CsAPX1‐RNAi group (Figure 5a,b). The results imply that *CsAPX1* serves as a susceptibility gene in response to *C*Las infection. Notably, APX activity was enhanced in citrus plants overexpressing *CLas0185*, and not changed by *C*Las infection (Figure 5c). Overexpression of *CsMsrB1* elevated APX activity in citrus plants, as well as in *C*Las0185‐OE rootlets, while *CsMsrB1*‐silencing decreased its activity in both backgrounds (Figure 5d,e). We propose a working model that the effector promotes HLB development by inhibiting ROS‐mediated plant immunity (Figure 5f). ROS generation increases in HLB‐diseased citrus plants. *C*Las0185 is secreted in *C*Las‐accumulated sieve cells, which enhances CsMsrB1 abundance, resulting in the reduction of ROS accumulation via elevating APX activity.

## 3 DISCUSSION

Prior studies revealed that *C*Las infects root during the initiation of colonization, and mainly accumulates in sieve cells where plant immune responses are inhibited (Bernardini et al., 2022; Johnson et al., 2014). Therefore, research on the attempts of the pathogen to bypass plant defence is necessary for the development of effective management strategies. In this study, we identified a *C*Las effector, CLas0185, that is highly expressed in rootlets and suppresses plant defence responses. Using the effector as a probe, we determined CsMsrB1 to be a target, which decreases H_2_O_2_ accumulation by enhancing enzymatic antioxidant activity, thereby offering mechanistic insights into the virulence strategies taken by *C*Las0185 to depress citrus innate immunity and promote HLB progression.

Redox homeostasis is associated with ageing and protein functions. Its dysregulation causes severe human diseases (Lee et al., 2017; Reiterer et al., 2019). Methionine (Met) belongs to the group of amino acids most susceptible to oxidation, and a diastereomeric mixture of Met‐*S*‐O and Met‐*R*‐O is formed in this reaction, leading to dysfunctional proteins (Le et al., 2018; Vogt, 1995). They can be reversed by two distinct Msr subfamilies, namely MsrA and MsrB, corresponding to different forms of Met‐O. Despite displaying a similar physiological function, the two subfamily proteins do not share homology at the primary sequence level or at the structural level. Due to the protective role in repairing oxidized proteins, Msr family proteins have been well studied in medicine (Moskovitz, 2005; Reiterer et al., 2019). The role of Msr proteins in plant physiology is receiving increasing attention, and progress has been made to dissect their functions in response to abiotic stresses. For instance, *Arabidopsis* root‐abundant *MsrB* genes are involved in tolerance to oxidative stress (Li et al., 2012). Ectopic expression of *ZmMsrB1* from *Z*. *mays* enhances salinity stress tolerance in *Arabidopsis* (Wang et al., 2022). By contrast, Msr functions implicated in biotic stresses are more complicated. The expression of *Msr* genes is manipulated in response to pathogen infection (Sadanandom et al., 2000). A pepper (*Capsicum annuum*) *CaMsrB2* gene activates defence against pathogen attack (Oh et al., 2010). *MsrB8* is required for defence against avirulent pathogens in *Arabidopsis* (Roy & Nandi, 2017). SlMsrB5 contributes to tomato fruit defence response against *Botrytis cinerea* induced by methyl jasmonate (Fu et al., 2023). Notably, NIa‐pro of papaya ringspot virus interacts with papaya PaMsrB1 to scavenge ROS caused by virus infection (Gao et al., 2012). Similarly, *C*Las0185 binding with CsMsrB1 inhibits H_2_O_2_ accumulation, and their interaction contributes to *C*Las proliferation. As Msr family proteins fulfil key signalling roles in the transmission of ROS‐related information (Chu et al., 2016; Jacques et al., 2015; Sun et al., 2016), further exploration could focus on the effect of *C*Las0185–CsMsrB1 interaction on ROS‐mediated signalling transduction.

Previous studies verified the interplay between Msr proteins and enzymatic antioxidants. For instance, APX1 activity can be enhanced by MsrB2, which regulates the redox state of banana fruit during ripening and senescence (Xiao et al., 2021). Among these antioxidants, APX has the highest affinity for H_2_O_2_ (Huang et al., 2018). It has been determined that *APX* expression and protein activity are manipulated in response to pathogen infection (Pérez‐Clemente et al., 2014; Zhang, Song, et al., 2023), while the reduction of APX activity results in ROS accumulation, thereby enhancing plant innate immunity (Chandrashekar & Umesha, 2014; Fujiwara et al., 2016). A prior study reported that a prophage‐encoded effector from *C*Las targets CsAPX6 to facilitate *Xanthomonas citri* subsp. *citri* infection (Du et al., 2023). Here, CsAPX1 was determined to promote HLB. These results suggest that APX plays an important role in HLB pathogenesis. In this study, we found that the expression of the *C*Las effector CLas0185 and its host target CsMsrB1 can enhance APX activity and inhibit ROS accumulation. It has been established that ROS accumulation intensifies HLB symptoms, which can be mitigated through foliar sprays of antioxidants (Clark et al., 2020; Ma, Pang, et al., 2022). HLB symptoms were eliminated in 0185‐transgenic *C*. *sinensis*. Intriguingly, we determined that the reduction of ROS accumulation by *C*Las0185 contributes to *C*Las proliferation. Nevertheless, a decrease in bacterial titres is observed with this foliar application (Ma, Pang, et al., 2022). Hence, we suppose that *C*Las0185 uses multiple tactics to facilitate *C*Las pathogenicity.

Screened by Y2H assay, 48 candidate proteins were identified to interact with *C*Las0185, revealing a wide range of functions for the effector. Among them, protein disulphide‐isomerase plays a role in catalysing misfolded proteins, which contributes to plant resistance (Li et al., 2020). Histone acetylation confers a function on acetylated lysine residues of the core histones, facilitating plant adaption to different stresses (Kumar et al., 2021). Notably, candidates including heat shock 70 kDa protein, small ubiquitin‐related modifier, and constitutive photomorphogenesis protein regulate protein degradation (Berka et al., 2022; Ghimire et al., 2020; Ouyang & Frucht, 2021). In this study, we discovered that the amount of CsMsrB1 is enhanced in *C*Las0185‐transgenic *C*. *sinensis*, supposing that the *C*Las effector could block the degradation pathway of CsMsrB1. Further studies are needed to investigate the potential roles of *C*Las0185.

Successful infection entails complex defence/counterdefence interplay, which is reflected by numerous effectors predicted from *C*Las genome (Granato et al., 2020; Prasad et al., 2016). Previous work identified several effectors that elicit plant immune response, for instance, CLIBASIA_05315, AGH17470, and *C*Las4425 (Du et al., 2022; Pitino et al., 2016; Zhang, Wang, et al., 2023). Most of the identified *C*Las effectors, including CLIBASIA_00460, SDE15, CLIBASIA_03875, and SDE4405, suppress elicitor‐triggered cell death (Oh et al., 2022; Pang et al., 2020; Zhang et al., 2019, 2020). Moreover, 10 non‐classical *C*Las effectors have been characterized as cell death suppressors (Du et al., 2021). There is substantial evidence of antagonistic interactions between effector‐triggered immunity (ETI)‐suppressing and ‐eliciting effectors. For instance, two *Phyptophthora sojae* effectors PsCRN63/115 can perturb plant H_2_O_2_ homeostasis through direct interaction with catalases (Zhang et al., 2015). *Pseudomonas syringae* effector HopQ1a suppresses AvrPto1m‐mediated ETI (Martel et al., 2022). An effector–effector interaction has also been identified within *C*Las effectors, in which CLIBASIA_05330 inhibits ETI induced by SDE1 (Shen et al., 2022). *C*Las4425 has been identified as an elicitor, and its cell death‐inducing activity is dependent on Botrytis‐induced kinase 1 in *N*. *benthamiana*, which is a key player in ROS‐mediated plant immunity (Zhang, Wang, et al., 2023). Intriguingly, *C*Las4425‐triggered cell death can be suppressed by *C*Las0185. Further analysis on this countereffect would provide a growing understanding of coordinated effector activities.

To sum up, through characterization of a *C*Las effector, *C*Las0185, with the activity to suppress elicitor‐triggered cell death, we discovered CsMsrB1 as its binding protein in *C*. *sinensis*, and their interaction facilitated *C*Las infection. On the one hand, CsMsrB1, restoring the enzymatic activity of CsAPX1, scavenges H_2_O_2_ accumulation induced by *C*Las infection, which prevents ROS toxicity; on the other hand, it catalyses the reduction of Met‐O back to Met, which may impair ROS‐mediated plant defence. This study unveils the virulence strategy used by *C*Las0185 to depress host immunity.

## 4 EXPERIMENTAL PROCEDURES

All recombinant vectors were constructed using ClonExpress II One Step Cloning Kit (Vazyme) and were confirmed by Sanger sequencing. The strains and vectors are in Table S2; the primers used in the study are listed in Table S3. The origin of *C*Las strain was from Guangxi Province of China.

### 4.1 Plants, ACPs and growth conditions

*N*. *benthamiana* plants were grown in a greenhouse at 25°C with an 18 h light/6 h dark cycle. The citrus plants were cultured at 28°C. *C*Las‐infected mandarin orange plants (*Citrus reticulata*) were used for nucleotide extraction, as well as the source for graft inoculation. The epicotyl segments of *C*. *sinensis* ‘Wanjincheng’ were used for generation of transgenic citrus plants, and 1‐year‐old rough lemon plants (*Citrus jambhiri*) as rootstocks. cDNA of infected ACPs was from the National Navel Orange Engineering Research Center, Gannan Normal University, Ganzhou.

### 4.2 Plasmid construction

Genomic DNA was extracted from *C*Las‐infected *C*. *reticulata* plants; mature form of *CLas0185* (a 150 bp coding region without the signal peptide) was amplified. The *GUS* fragment (a 300 bp coding region) was amplified from vector pLGN. Total RNA was extracted from leaves of *C*. *sinensis*, and reverse transcribed into cDNA, from which full‐length of *CsMsrB1* (XM_006477914.4) and *CsAPX1* (XM_006488132.4) were amplified.

For *Agrobacterium*‐mediated transient expression in *N*. *benthamiana*, the coding sequences of *CLas0185*, *CLas0185‐GUS*, and *BAX* were amplified with PCR or overlap PCR, and cloned into PVX, PVX‐HA, and PVX‐FLAG, respectively. PVX‐HA, PVX‐FLAG, PVX‐GFP‐HA, and PVX‐*C*Las4425‐FLAG were constructed in the laboratory. For the Y2H assay, *CLas0185* and genes encoding enzymatic antioxidants, including *CsAPXs*, were cloned into the bait vector pGBKT7‐BD (Clonetech). *CsMsrB1* was inserted into the prey vector pGADT7‐AD (Clonetech) to generate AD‐CsMsrB1. For the LCI assay, the plasmids of *C*Las0185‐nLuc, CsAPX1‐nLuc, HA‐nLuc, CsMsrB1‐cLuc, and HA‐cLuc were constructed. NbGAPC‐nLuc and NbATG3‐cLuc were constructed in the laboratory. For the pull‐down assay, the plasmids of GST‐*C*Las0185, GST‐CsAPX1, and His‐CsMsrB1 were generated. For *Agrobacterium*‐mediated transient expression in *C*. *sinensis*, the coding sequences of *CsAPX1* and *CsMsrB1* were cloned into pLGN‐HA and pLGN‐FLAG, respectively. pLGN‐GUS‐HA and pLGN‐GUS‐FLAG were constructed. pLGN‐HA and pLGN‐FLAG were constructed in the laboratory. To prepare the stable overexpression plasmids, the amplified fragments of *CLas0185*, *CsMsrB1*, and *CsAPX1* were cloned into pLGN. To obtain RNA interference plasmids, 300‐bp fragments in *CsMsrB1* and *CsAPX1* were selected and inserted into the pGN vector between the AscI and SwaI restriction sites in the antisense orientation and between the BamHӀ and SalӀ restriction sites in the sense orientation to generation a hairpin. For the virus‐induced gene silencing (VIGS) assay, the 300‐bp fragments in *CsMsrB1*, *CsAPX1*, and *GUS* were cloned into pCLBV.

### 4.3 Transient expression in planta

For transient expression in *N*. *benthamiana*, *Agrobacterium tumefaciens* GV3101 (pJIC SA_Rep) was used. BAX and *C*Las effector *C*Las4425 (Li et al., 2015; Zhang, Wang, et al., 2023) served as positive controls that induce cell death. To determine the effect of *C*Las0185 on ETI, *N*. *benthamiana* leaves were first infiltrated with recombinant strains of *A*. *tumefaciens* carrying *C*Las0185 or GFP, and the elicitor was injected in the same regions at 1 dpi. The leaves were stained using 3,3′‐diaminobenzisine (DAB) staining at 3 dpi and trypan blue staining at 5 dpi (Li et al., 2019), and symptoms were observed and photographed at 7 dpi. The experiments were repeated three times.

For transient expression in *C*. *sinensis*, *A*. *tumefaciens* EHA105 was used. The suspension expressing target genes and GUS (as control) were injected in different sides of the same leaf on opposite sides of the main vein (Du et al., 2022). The experiments were repeated twice.

### 4.4 Immunoblotting

Protein was extracted from infiltrated leaves using Plant Protein Extraction Kit (Beijing Solarbio Science & Technology Co., Ltd) (Tang et al., 2023). Total proteins were separated by 12.5% SDS‐PAGE (Epizyme), and the protein samples were transferred to a polyvinylidene difluoride (PVDF) membrane. Primary monoclonal antibodies (Proteintech) were diluted to 1:500 while horseradish peroxidase‐conjugated goat anti‐mouse IgG was used as a secondary antibody at a dilution of 1:10,000.

### 4.5 Nucleotide extraction and detection analysis

Total DNA was extracted from diseased‐citrus midribs (0.1 g) using Biospin Omini Plant Genomic DNA Extraction Kit (BioFlux), following the manufacturer's instruction. Quantification of *C*Las was performed using qPCR assay. *C*Las housekeeping gene *CLasgyrA* was detected, and 18S rRNA of *C*. *sinensis* was used as the endogenous control. BlastTaq 2× qPCR MasterMix (ABM) was used for qPCR amplification. The experiment contained three independent biological replicates, and three technical repeats were performed. The bacterial titres (*C*Las cells per μg of citrus DNA) were quantified with qPCR assay that was described by Zou et al. (2016): $C\mathrm{Las}\;\text{titre}=10^{\left(10.624–0.2718\times {\mathrm{Ct}}_{\text{CLasgyrA}}\right)}/10^{\left(4.0531–0.2749\times Ct_{18\text{SrRNA}}\right)}\times 1000$.

TaqMan qPCR was applied to quantify bacterial titre in citrus rootlets. DNA was extracted from hairy roots at 3 months after transformation, and the concentrations were adjusted to 10 ng/μL. The PCR system consisted of 16S rDNA primer HLBasr/ HLBasf, HLBp, DNA template, and TaqProbe 2× qPCR (ABM) in a total volume of 20 μL. The experiment contained four independent biological replicates, and three technical repeats were performed. The bacterial titres (*C*Las cell per μg of citrus DNA) were quantified with qPCR assay as was described by Li et al. (2006): $C\mathrm{Las}\;\text{titre}=10^{\left(12.715-Ct_{16S\;\text{rRNA}}\times 0.3264\right)}/0.01$.

Total RNA was extracted from citrus leaves using RNAiso Plus (Takara). In a 20‐μL volume, 1 μg of total RNA was reverse transcribed with All‐In‐One 5× RT MasterMix (ABM) following the manufacturer's instructions. RT‐qPCR assays were conducted to analyse gene transcript level. *C*. *sinensis* housekeeping gene *CsGAPDH* was used as the internal reference. The reaction was performed in a 10‐μL volume. The experiments contained three independent biological replicates, and three technical repeats were performed. The 2^−ΔΔ*C*t^ method was used for the determination of relative gene transcription (Livak & Schmittgen, 2001).

### 4.6 Plant transformation and *C*Las inoculation

To generate *C*Las0185 transgenic citrus plants, the recombinant plasmid was transferred into *A*. *tumefaciens* EHA105. *Agrobacterium*‐mediated transformation of etiolated epicotyl segments of Wanjincheng was as previously described (Peng et al., 2015). Transgenic lines were confirmed by GUS staining, and the buds without transformation served as the WT control. They were micrografted onto 1‐month‐old Wanjincheng rootstock seedlings. The resulting plantlets were further grafted onto *C. jambhiri* rootstock in a greenhouse. Transgenic plants were validated by PCR and RT‐qPCR at the RNA level. For HLB pathogenicity assay, citrus plants were inoculated by stems of *C*Las‐infected *C. reticulata* plants. Each transgenic line represents an independent transgenic event. Leaf samples were collected monthly. *C*Las populations were determined with qPCR.

To conduct the transformation of citrus hairy roots, the recombinant plasmids were transferred into *A*. *rhizogenes* K599. For *CsMsrB1* transformation, the stem sections were removed from HLB‐diseased *C*Las0185‐transgenic Wanjingchen. For *CsAPX1* transformation, the stem sections were detached from wild‐type Wanjingchen infected by *C*Las. The details of performance were described previously (Ma, Meng, et al., 2022). The rootlets were collected at 3 mpi for PCR and GUS staining. After the determination of transgenic rootlets, the expression levels of corresponding genes and *C*Las titres were confirmed by RT‐qPCR and TaqMan qPCR, respectively.

### 4.7 Y2H assay

A cDNA library from *C*. *sinensis* was constructed to screen for proteins interacting with *C*Las0185 using the GAL4 system as described in the BD Matchmaker Library Construction and Screening Kits User Manual (Clontech). Using BD‐*C*Las0185 as the bait, the interaction protein of *C*Las0185 was screened in *Saccharomyces cerevisiae* as described by Shi, Yang, et al. (2023). The full‐length coding sequences of the candidate interacting preys were cloned into AD and retested with BD‐*C*Las0185 in SD/−Leu/−Trp/ (DDO), SD/−Leu/−Trp/−Ade/−His (QDO) containing 40 μg/mL of X‐α‐gal. AD‐CsMsrB1 was co‐transformed with enzymatic antioxidants‐inserted BD vectors to evaluate their inter‐relationship. The experiments were repeated twice.

### 4.8 LCI assay

LCI assays were performed as described (Zhou et al., 2018). All combinations tested were agro‐infiltrated into leaves of *N*. *benthamiana*. The leaves were detached at 3 dpi, sprayed with 1 mM luciferin, and observed under a chemiluminescence apparatus (Bio‐Rad). The pictures were taken 1 min after exposure. The experiments were repeated twice.

### 4.9 Pull‐down assay

The pull‐down assays were performed as described previously (Pang et al., 2020). Proteins were generated in *Escherichia coli* Rosetta 2 (DE3) (Weidi), and were purified using glutathione‐ or Ni‐NTA‐sepharose beads (Beyotime) following the manufacturer's instructions. After 1 h incubation, the beads were washed five times, then boiled in 5× SDS loading buffer (Epizyme). The proteins were separated by SDS‐PAGE for immunoblot assays. The experiments were repeated twice.

### 4.10 CLBV‐mediated VIGS in *C*. *sinensis*

Plasmids containing derivatives of the binary CLBV vectors, namely CLBV:*CsAPX1*, CLBV:*CsMsrB1*, and CLBV:*GUS* (as control), were transferred into *A*. *tumefaciens* GV3101. The assays were conducted following the procedure applied by Zhao et al. (2020). *Agrobacterium* cells harbouring CLBV constructs were cultured in Luria Bertani broth to an OD_600_ = 1.0, and resuspended in the infiltration buffer. One‐week‐old Wanjincheng seedlings were infiltrated in suspension by the vacuum method. After infiltration, the seedlings were growth in darkness for 2 days and then moved to a growth chamber for 1 month. Virus infection and silencing efficiency were determined with RT‐PCR and RT‐qPCR, respectively.

### 4.11 Oxidation and reduction of CsAPX1

The assays were conducted following the procedure applied by Xiao et al. (2021). CsAPX1 protein (1 mg) was incubated with H_2_O_2_ (10 mM) at 22°C for 3 h in 50 mM phosphate‐buffered saline (pH 7.0). H_2_O_2_ was removed by centrifugal filter units (Merck Millipore). Oxidized CsAPX1 (2 μM O‐CsAPX1) was then incubated with 2 μM CsMsrB1 and 10 mM dithiothreitol (DTT) at 37°C for 3 h. DTT was removed using centrifugal filter units. The proteins were collected and analysed by immunoblot, which were further sent to Shenzhen Wininnovate Biotechnology Co., Ltd for determination of Met‐O content through LC–MS/MS. The experiments were repeated twice.

### 4.12 Measurement of physiological parameters

Assay kits (Beijing Solarbio Science & Technology Co., Ltd) were applied to measure H_2_O_2_ and APX activity, and determined by spectrophotometry (Li et al., 2024; Yang et al., 2022). For in vivo analysis, each sample (0.1 g) was finely ground with liquid nitrogen, and the content of H_2_O_2_ as well as the APX enzymatic activity were measured and assayed following the manufacturer's instructions. For in vitro analysis, 20 μg of native CsAPX1, O‐CsAPX1, and CsMsrB1‐reduced CsAPX1 were prepared for APX activity measurement. The experiments were repeated twice.

### 4.13 Statistical analysis

Statistical analysis was performed with SPSS Statistics 19 (IBM). Data were analysed using Fisher's LSD test (*p <* 0.05 as the level of significance) or Student's *t* test (*p < 0.05; **p < 0.01; ***p < 0.001). Each value was the mean ± *SD* of three independent replicates.

## CONFLICT OF INTEREST STATEMENT

The authors declare that they have no conflict of interest.

## Supporting information


**FIGURE S1.** Generation of *C*Las0185‐transgenic *Citrus sinensis* ‘Wanjincheng’ (0185‐OE). (a) Structure of the pLGN‐*C*Las0185 applied for the overexpression assay. (b, c) Identification of transgenic plants with PCR (b), β‐glucuronidase (GUS) histochemical staining (c). M, DNA, marker; 0185‐OE#, transgenic lines expressing *CLas0185*; WT, wild‐type control. Scale bar: 9 mm. (d) Reverse transcription‐quantitative PCR to confirm the expression of *CLas0185* in the transgenic citrus plants. (e) Phenotypes of WT and 0185‐OE. Scale bar: 10 cm. (f) Internode lengths of citrus plants were measured. Data in (d, f) represent mean ± *SD*. Different letters indicate significant differences determined by Student’s *t* test (*p* < 0.05; *n* = 3). The experiments comprised three independent biological replicates, and three technical repeats were performed.

**FIGURE S2.** Phylogeny and sequence alignment of Methionine sulphoxide reductase (Msr) gene family. (a) Phylogenetic analysis of Msr homologues within *Citrus sinensis* (6), *Nicotiana tabacum* (10), *Musa acuminata* (6), and *Zea mays* (3). Plant species and their corresponding symbols are listed on the right top of the figure. Neighbour‐joining method was applied to generate the phylogeny with 1000 bootstrap replicates. Bootstrap values are indicated at each node. Scale bar: 0.20. According to amino sequence identity, Msr homologues were divided into MsrA and MsrB groups, and six subgroups (I–VI). The resulting phylogeny was visualized using MEGA 11. (b) Alignment of amino acid sequences of CsMsrB1, MaMsrB1, NtMsrB1, NtMsrB, and ZmMsrB. The alignment was performed using GeneDoc with default parameters. CsMsrB1 is highlighted in red. Black shading indicates 100% similarity across sequences. The SelR domain indicated by the red arrow plays a role in maintaining intracellular redox homeostasis via reducing the *R*‐form of methionine sulphoxide back to methionine.

**FIGURE S3.** Generation of genetic transformation hairy roots overexpressing/silencing *CsMsrB1*. (a) Generation of transgenic *Citrus sinensis* ‘Wanjincheng’ overexpressing/silencing *CsMsrB1*. Structures of the pLGN‐CsMsrB1 applied for the overexpression assay, and pGN‐CsMsrB1‐RNAi for gene silencing. *CsMsrB1*‐overexpressing (CsMsrB1‐OE) and *CsMsrB1*‐silenced (CsMsrB1‐RNAi) transgenic citrus hairy roots were generated using *Agrobacterium rhizogenes*‐mediated transformation, which developed from ‘*Candidatus* Liberibacter asiaticus’ (*C*Las)‐infected 0185‐OE stem sections. The resulting transgenic plants were verified with PCR. M, DNA marker; 0185‐OE, the negative control; 0185‐OE + CsMsrB1‐OE#, transgenic lines expressing *CsMsrB1* in a 0185‐OE background. (b) Phenotypes of *A*. *rhizogenes‐*induced hairy root. Scale bar: 10 mm. (c, d) Relative expression levels of *CsMsrB1*. Transcript levels measured with reverse transcription‐quantitative PCR were normalized to levels in *C*Las‐infected 0185‐OE using the *CsGAPDH* as endogenous control. The differences were analysed using Student’s *t* test (**p < 0.01; ***p < 0.001, *n* = 4).

**FIGURE S4.** Transient expression and citrus leaf blotch virus (CLBV)‐induced gene silencing (VIGS) of *CsMsrB1* in *Citrus sinensis* ‘Wanjincheng’. (a) Immunoblot analysis. CsMsrB1 and β‐glucuronidase (GUS) C‐terminally fused with FLAG were expressed in citrus leaves through *Agrobacterium* infiltration. Protein was extracted at 3 days post‐infiltration (dpi) and was verified by immunoblotting with an anti‐FLAG antibody, and equal loading of each sample is confirmed with immunoblot of RuBisCO. Mock represents the wild‐type (WT) negative control. (b) Reverse transcription PCR was used to determine the fragment insertion. CLBV:*GUS_*1–7 represent CLBV:*GUS* inoculated citrus plants, and CLBV:*CsMsrB1*_1–5 represent CLBV:*CsMsrB1* inoculated citrus plants. ‘−’ represents WT as the negative control. (c, d) Relative expression levels of *CsMsrB1* in Wanjincheng. Transcripts levels of *CsMsrB1* measured with reverse transcription‐quantitative PCR were normalized to levels in GUS‐OE/CLBV:*GUS* control using the *CsGAPDH* as endogenous control. The differences were analysed using Student’s *t* test (**p < 0.01; ***p < 0.001, *n* = 3).

**FIGURE S5.** Validation of the interactions between CsMsrB1 and enzymatic antioxidants using yeast two‐hybrid (Y2H) assay. Glutathione reductase (GR) (XP_006493268.2), monodehydroascorbate reductase (MDAR) (XP_006476500.2), SOD[Fe] (XP_006485042.1), SOD[Cu‐Zn] (XP_006471806.2), ascorbate peroxidase 1 (APX1, XP_015388868.1), and catalase 1 (CAT1, XP_015388868.1) served as candidates. Serial 10‐fold dilutions of co‐transformed yeast cells on double‐dropout (DDO) and quadruple‐dropout (QDO) + X‐α‐gal are shown. The experiments were performed twice, with similar results.

**FIGURE S6.** Sequence analysis of ascorbate peroxidases (APXs) and the inter‐relationship between CsMsrB1 and CsAPXs. (a) Phylogenetic analysis of APX homologues within *Citrus sinensis* (6), *Nicotiana tabacum* (3), *Arabidopsis thaliana* (6), and *Zea mays* (7). Plant species and their corresponding symbols are listed on the right top of the figure. Neighbour‐joining method was applied to generate the phylogeny with 1000 bootstrap replicates. Bootstrap values are indicated at each node. Scale bar: 0.20. According to amino sequence identity, APX homologues were divided into five clusters (I–V). (b) Alignment of amino acid sequences of CsAPXs. Black shading indicates 100% similarity across sequences. (c) The interactions between CsMsrB1 and CsAPXs were verified with pairwise yeast two‐hybrid assays. Serial 10‐fold dilutions of co‐transformed yeast cells on double‐dropout (DDO) and quadruple‐dropout (QDO) + X‐α‐gal are shown. The experiments were performed twice, with similar results.

**FIGURE S7.** Transient expression and virus‐induced gene silencing of *CsAPX1* in *Citrus sinensis*. (a) Immunoblot analysis. CsAPX1 and GUS C‐terminally fused with HA were expressed in citrus leaves through *Agrobacterium* infiltration. Protein was extracted at 3 days post‐infiltration, and was verified by immunoblotting with an anti‐HA antibody, and equal loading of each sample is confirmed by immunoblot of RuBisCO. Mock represents the wild‐type (WT) negative control. (b) Reverse transcription‐PCR was used to determine the fragment insertion. CLBV:*GUS_*1–7 represent CLBV:*GUS* inoculated citrus plants, and CLBV:*CsAPX1*_1–5 represent CLBV:*CsAPX1* inoculated citrus plants. ‘−’ represents WT as the negative control. CLBV:*GUS* served as the control for both CLBV:*CsMsrB1* and CLBV:*CsAPX1*, thereby the figure of CLBV:*GUS* agarose gel electrophoresis is the same as that used in Figure S4b. (c, d) Relative expression levels of *CsAPX1* in Wanjincheng. Transcripts levels of *CsAPX1* measured with reverse transcription‐quantitative PCR were normalized to levels in GUS‐OE/CLBV:*GUS* control using the *CsGAPDH* as endogenous control. The differences were analysed using Student’s *t* test (**p < 0.01, *n* = 3).

**FIGURE S8.** Generation of genetic transformation hairy roots overexpressing/silencing *CsAPX1* in huanglongbing‐diseased citrus plants. (a) Generation of transgenic hairy roots overexpressing/silencing *CsAPX1*. Structures of the pLGN‐CsAPX1 applied for the overexpression assay, and pGN‐CsAPX1‐RNAi for gene silencing. Identification of transgenic plants with PCR and β‐glucuronidase GUS) staining. M, DNA marker; WT, wild‐type control; CsAPX1‐OE#, transgenic lines expressing *CsAPX1*. (b) Phenotypes of *Agrobacterium rhizogenes‐*induced hairy root. Scale bar: 10 mm. (c, d) Relative expression levels of *CsAPX1* in citrus plants. Transcripts levels of *CsAPX1* measured with reverse transcription‐quantitative PCR were normalized to levels in ‘*Candidatus* Liberibacter asiaticus’‐infected WT using the *CsGAPDH* as endogenous control. The differences were analysed using Student’s *t* test (**p < 0.01; ***p < 0.001, *n* = 4).

**TABLE S1.** Candidate proteins that interact with *C*Las0185 via yeast two‐hybrid assay.

**TABLE S2.** Strains and plasmids used in this study.

**TABLE S3.** Primers used in this study.

## Data Availability

All relevant data used to support the findings of this study are available from the corresponding author upon request.
