# Supplementary material for: Effector CLas0185 targets methionine sulphoxide reductase B1 of Citrus sinensis to promote multiplication of ‘Candidatus Liberibacter asiaticus’ via enhancing enzymatic activity of ascorbate peroxidase 1
Source: Mol Plant Pathol. 2024 Aug 31;25(9):e70002. doi: 10.1111/mpp.70002 (PMC11365454; doi:10.1111/mpp.70002)
Supplement: Supplementary file 1 — FIGURE S1. Generation of CLas0185‐transgenic Citrus sinensis ‘Wanjincheng’ (0185‐OE). (a) Structure of the pLGN‐CLas0185 applied for the overexpression assay. (b, c) Identification of transgenic plants with PCR (b), β‐glucuronidase (GUS) histochemical staining (c). M, DNA, marker; 0185‐OE#, transgenic lines expressing CLas0185; WT, wild‐type control. Scale bar: 9 mm. (d) Reverse transcription‐quantitative PCR to confirm the expression of CLas0185 in the transgenic citrus plants. (e) Phenotypes of WT and 0185‐OE. Scale bar: 10 cm. (f) Internode lengths of citrus plants were measured. Data in (d, f) represent mean ± SD. Different letters indicate significant differences determined by Student’s t test (p < 0.05; n = 3). The experiments comprised three independent biological replicates, and three technical repeats were performed. [file MPP-25-e70002-s009.docx]

**
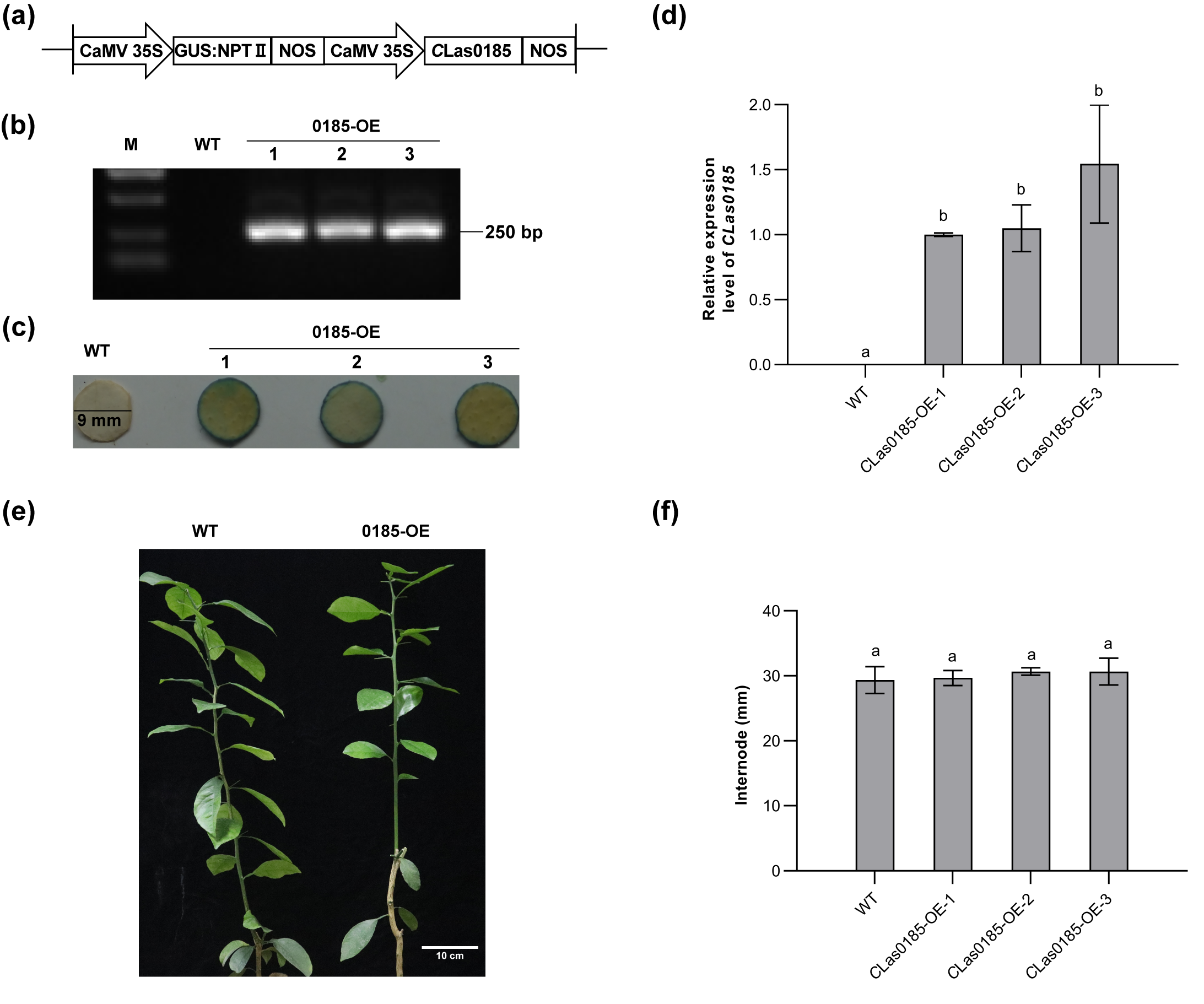
Figure S1.** Generation of *C*Las0185-transgenic Wanjincheng (*Citrus* *sinensis*) (0185-OE).

**(a)** Structure of the pLGN-*C*Las0185 applied for the overexpression assay. **(b, c)** Identification of transgenic plants with PCR **(b)**, β-glucuronidase (GUS) histochemical staining **(c)**. M, DNA marker; 0185-OE#, transgenic lines expressing *CLas0185*; WT, wild-type control. Scale bar: 9 mm. **(d)** qRT-PCR to confirm the expression of *CLas0185* in the transgenic citrus plants. **(e)** Phenotypes of WT and 0185-OE. Scale bar: 10 cm. **(f)** Internode lengths of citrus plants were measured. Data in **(d, f)** represent mean**±**SD. Different letters indicate significant differences determined by the Student’s *t*-test (*P*<0.05; n=3). The experiments comprised three independent biological replicates, and three technical repeats were performed.
