# Supplementary material for: Effector CLas0185 targets methionine sulphoxide reductase B1 of Citrus sinensis to promote multiplication of ‘Candidatus Liberibacter asiaticus’ via enhancing enzymatic activity of ascorbate peroxidase 1
Source: Mol Plant Pathol. 2024 Aug 31;25(9):e70002. doi: 10.1111/mpp.70002 (PMC11365454; doi:10.1111/mpp.70002)

**Figure S2.** Phylogeny and sequence alignment of Metthionine sulfoxide reductase (Msr) gene family. **(a)** Phylogenetic analysis of Msr homologous within *Citrus sinensis*, *Nicotiana* *tobaccum*, *Musa acuminate*, and *Zea mays*. Plant species and their corresponding symbols are listed on the right top of the figure. Neighbor-Joining method was applied to generate the phylogeny with 1 000 bootstrap replicates. Bootstrap values are indicated at each node. Scale bar: 0.20. According to amino sequence identity, Msr homologs were divided into MsrA and MsrB groups, and six subgroups (I-VI). The resulting phylogeny was visualized using MEGA 11. **(b)** Alignment of amino acid sequences of CsMsrB1, MaMsrB1, NtMsrB1, NtMsrB and ZmMsrB. The alignment was performed using GeneDoc with default parameters. CsMsrB1 was highlighted in red. Black shading indicates 100% similarity across sequences. The SelR domain indicated by the red arrow, plays a role in maintaining intracellular redox homeostasis via reducing the *R*-form of methionine sulfoxide back to methionine


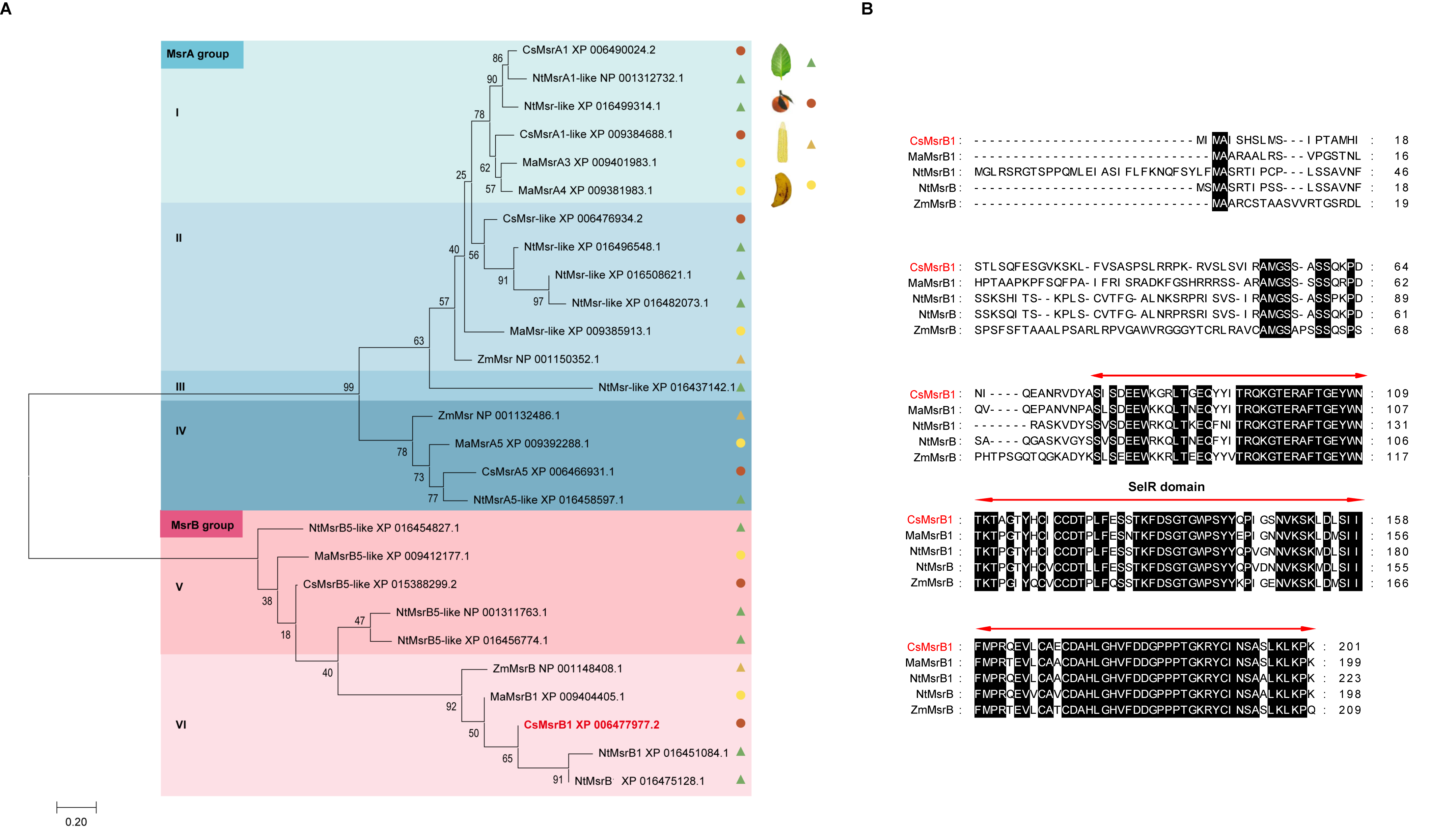

Supplement: Supplementary file 2 — FIGURE S2. Phylogeny and sequence alignment of Methionine sulphoxide reductase (Msr) gene family. (a) Phylogenetic analysis of Msr homologues within Citrus sinensis (6), Nicotiana tabacum (10), Musa acuminata (6), and Zea mays (3). Plant species and their corresponding symbols are listed on the right top of the figure. Neighbour‐joining method was applied to generate the phylogeny with 1000 bootstrap replicates. Bootstrap values are indicated at each node. Scale bar: 0.20. According to amino sequence identity, Msr homologues were divided into MsrA and MsrB groups, and six subgroups (I–VI). The resulting phylogeny was visualized using MEGA 11. (b) Alignment of amino acid sequences of CsMsrB1, MaMsrB1, NtMsrB1, NtMsrB, and ZmMsrB. The alignment was performed using GeneDoc with default parameters. CsMsrB1 is highlighted in red. Black shading indicates 100% similarity across sequences. The SelR domain indicated by the red arrow plays a role in maintaining intracellular redox homeostasis via reducing the R‐form of methionine sulphoxide back to methionine. [file MPP-25-e70002-s011.docx]
