# Supplementary material for: Effector CLas0185 targets methionine sulphoxide reductase B1 of Citrus sinensis to promote multiplication of ‘Candidatus Liberibacter asiaticus’ via enhancing enzymatic activity of ascorbate peroxidase 1
Source: Mol Plant Pathol. 2024 Aug 31;25(9):e70002. doi: 10.1111/mpp.70002 (PMC11365454; doi:10.1111/mpp.70002)
Supplement: Supplementary file 3 — FIGURE S3. Generation of genetic transformation hairy roots overexpressing/silencing CsMsrB1. (a) Generation of transgenic Citrus sinensis ‘Wanjincheng’ overexpressing/silencing CsMsrB1. Structures of the pLGN‐CsMsrB1 applied for the overexpression assay, and pGN‐CsMsrB1‐RNAi for gene silencing. CsMsrB1‐overexpressing (CsMsrB1‐OE) and CsMsrB1‐silenced (CsMsrB1‐RNAi) transgenic citrus hairy roots were generated using Agrobacterium rhizogenes‐mediated transformation, which developed from ‘Candidatus Liberibacter asiaticus’ (CLas)‐infected 0185‐OE stem sections. The resulting transgenic plants were verified with PCR. M, DNA marker; 0185‐OE, the negative control; 0185‐OE + CsMsrB1‐OE#, transgenic lines expressing CsMsrB1 in a 0185‐OE background. (b) Phenotypes of A. rhizogenes‐induced hairy root. Scale bar: 10 mm. (c, d) Relative expression levels of CsMsrB1. Transcript levels measured with reverse transcription‐quantitative PCR were normalized to levels in CLas‐infected 0185‐OE using the CsGAPDH as endogenous control. The differences were analysed using Student’s t test (**p < 0.01; ***p < 0.001, n = 4). [file MPP-25-e70002-s007.docx]

**
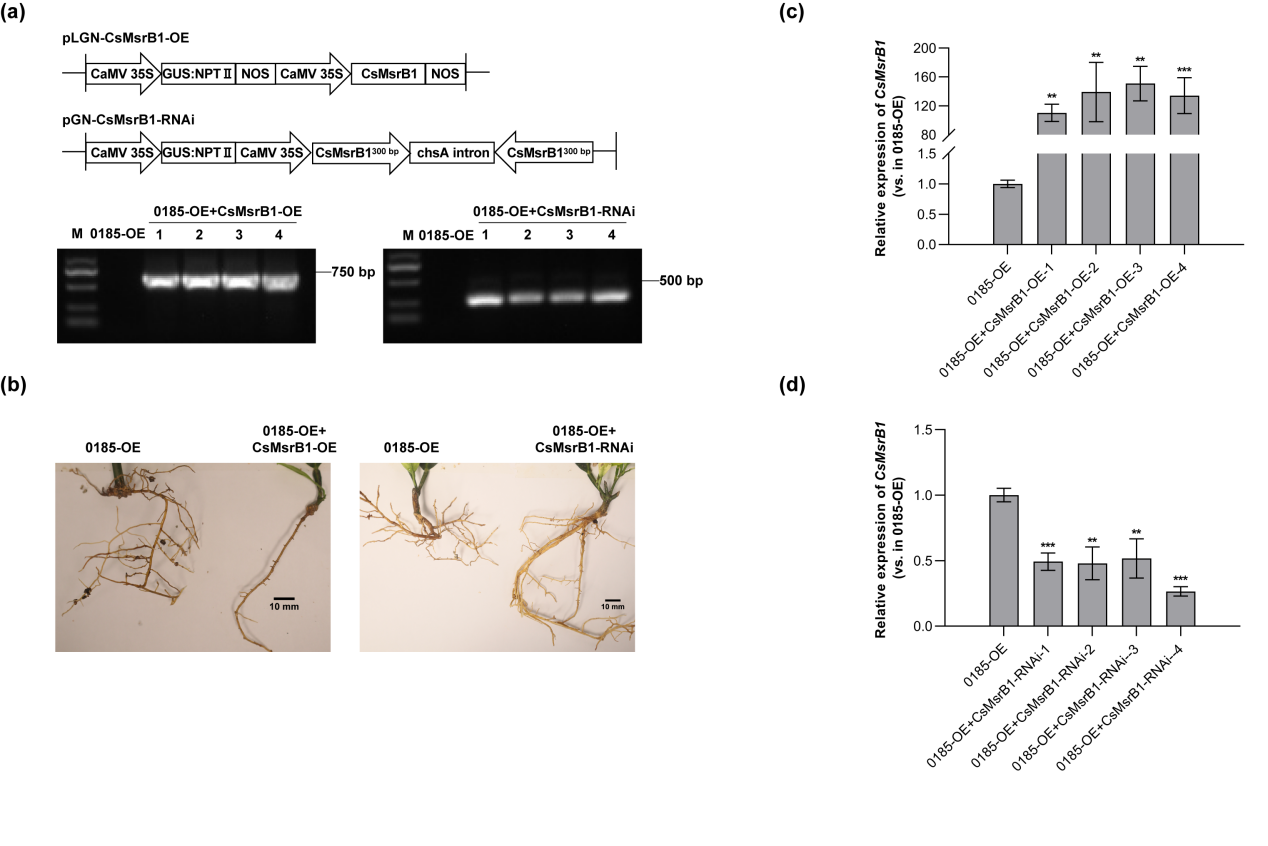
Figure S3.** Generation of genetic transformation hairy roots overexpressing/silencing *CsMsrB1*.

**(a)** Generation of transgenic Wanjincheng (*C*. *sinensis*) overexpressing/silencing *CsMsrB1*. Structures of the pLGN-CsMsrB1 applied for the overexpression assay, and pGN-CsMsrB1 for gene silencing. *CsMsrB1*-overexpressing (CsMsrB1-OE) and *CsMsrB1*-silencing (CsMsrB1-RNAi) transgenic citrus hairy roots were generated using *Agrobacterium rhizogenes*-mediated transformation, which developed from *C*Las-infected 0185-OE stem sections. The resulting transgenic plants were verified with PCR. M, DNA marker; 0185-OE, the negative control; 0185-OE+CsMsrB1-OE#, transgenic lines expressing *CsMsrB1* in a 0185-OE background. **(b)** Phenotypes of *A*. *rhizogenes-*induced hairy root. Scale bar: 10 mm. **(c, d)** Relative expression levels of *CsMsrB1*. Transcripts levels measured with qRT-PCR were normalized to levels in *C*Las-infected 0185-OE using the *CsGAPDH* as endogenous control. The differences were analyzed using Student’s *t*-test (***P*<0.01, ****P*<0.001; n=4).
