# Supplementary material for: Effector CLas0185 targets methionine sulphoxide reductase B1 of Citrus sinensis to promote multiplication of ‘Candidatus Liberibacter asiaticus’ via enhancing enzymatic activity of ascorbate peroxidase 1
Source: Mol Plant Pathol. 2024 Aug 31;25(9):e70002. doi: 10.1111/mpp.70002 (PMC11365454; doi:10.1111/mpp.70002)
Supplement: Supplementary file 4 — FIGURE S4. Transient expression and citrus leaf blotch virus (CLBV)‐induced gene silencing (VIGS) of CsMsrB1 in Citrus sinensis ‘Wanjincheng’. (a) Immunoblot analysis. CsMsrB1 and β‐glucuronidase (GUS) C‐terminally fused with FLAG were expressed in citrus leaves through Agrobacterium infiltration. Protein was extracted at 3 days post‐infiltration (dpi) and was verified by immunoblotting with an anti‐FLAG antibody, and equal loading of each sample is confirmed with immunoblot of RuBisCO. Mock represents the wild‐type (WT) negative control. (b) Reverse transcription PCR was used to determine the fragment insertion. CLBV:GUS_1–7 represent CLBV:GUS inoculated citrus plants, and CLBV:CsMsrB1_1–5 represent CLBV:CsMsrB1 inoculated citrus plants. ‘−’ represents WT as the negative control. (c, d) Relative expression levels of CsMsrB1 in Wanjincheng. Transcripts levels of CsMsrB1 measured with reverse transcription‐quantitative PCR were normalized to levels in GUS‐OE/CLBV:GUS control using the CsGAPDH as endogenous control. The differences were analysed using Student’s t test (**p < 0.01; ***p < 0.001, n = 3). [file MPP-25-e70002-s003.docx]

**
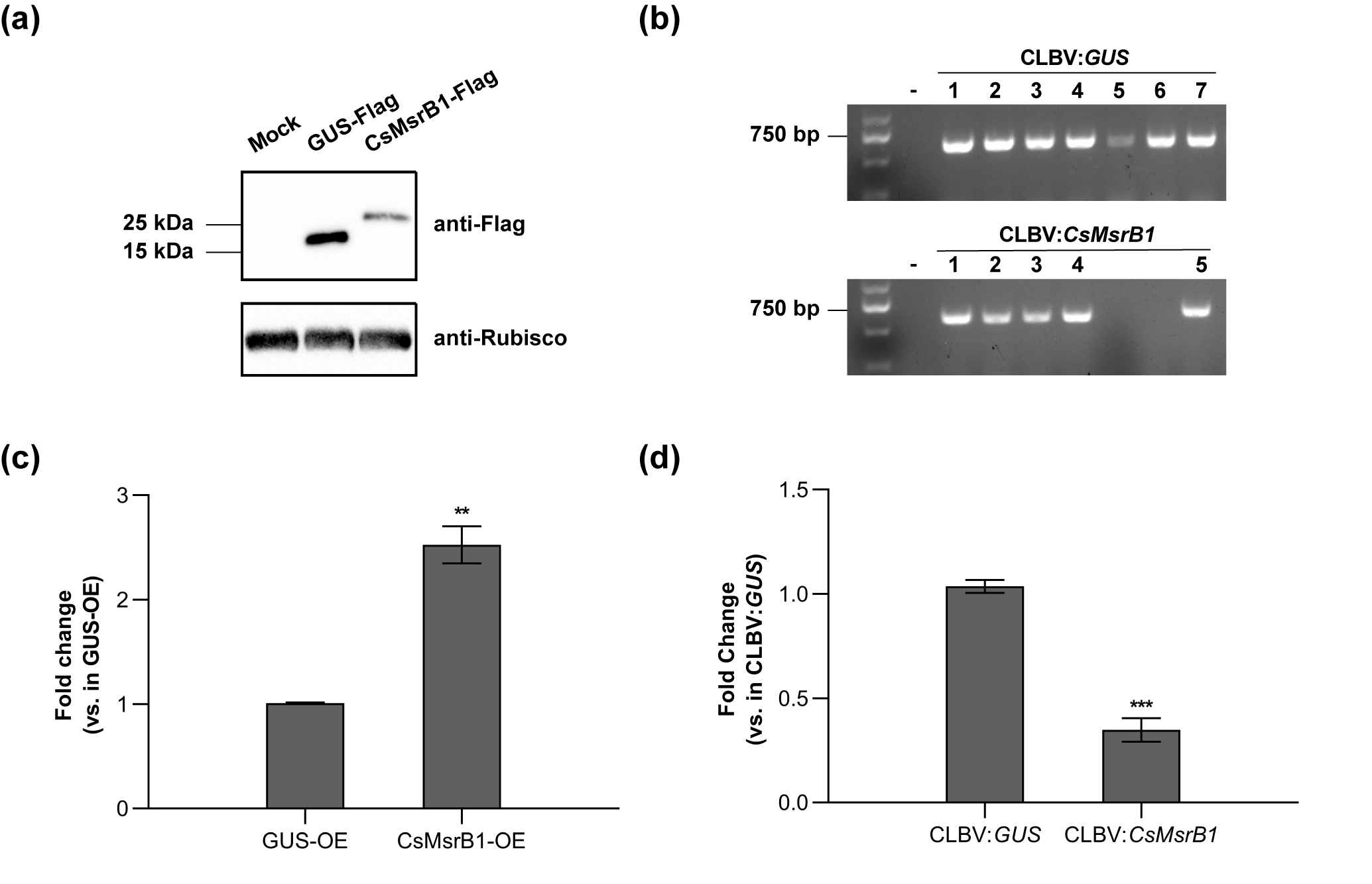
Figure S4.** Transient expression and citrus leaf blotch virus (CLBV)-induced gene silencing (VIGS) of *CsMsrB1* in Wanjincheng (*C*. *sinensis*).

**(a)** Immunoblot analysis. CsMsrB1 and GUS C-terminally fused with Flag were expressed in citrus leaves through agrobacterium infiltration. Protein was extracted at 3 days post-infiltration (dpi), and was verified by immunoblotting with a Flag antibody, and equal loading of each sample is confirmed with immunoblot of Rubisco. Mock represents the wild-type negative control. **(b)** RT-PCR was used to determined the fragment insertion. CLBV:*GUS_*1-7 represent CLBV:*GUS* inoculated citrus plants, and CLBV:*CsMsrB1*_1-5 represent CLBV:*CsMsrB1* inoculated citrus plants. ‘-’ represents WT as the negative control. **(c, d)** Relative expression levels of *CsMsrB1* in Wanjincheng. Transcripts levels of *CsMsrB1* measured with qRT-PCR were normalized to levels in GUS-OE/CLBV:*GUS* control using the *CsGAPDH* as endogenous control. The differences were analyzed using Student’s *t*-test (***P*<0.01, ****P*<0.001, n=3).
