# Supplementary material for: Effector CLas0185 targets methionine sulphoxide reductase B1 of Citrus sinensis to promote multiplication of ‘Candidatus Liberibacter asiaticus’ via enhancing enzymatic activity of ascorbate peroxidase 1
Source: Mol Plant Pathol. 2024 Aug 31;25(9):e70002. doi: 10.1111/mpp.70002 (PMC11365454; doi:10.1111/mpp.70002)
Supplement: Supplementary file 5 — FIGURE S5. Validation of the interactions between CsMsrB1 and enzymatic antioxidants using yeast two‐hybrid (Y2H) assay. Glutathione reductase (GR) (XP_006493268.2), monodehydroascorbate reductase (MDAR) (XP_006476500.2), SOD[Fe] (XP_006485042.1), SOD[Cu‐Zn] (XP_006471806.2), ascorbate peroxidase 1 (APX1, XP_015388868.1), and catalase 1 (CAT1, XP_015388868.1) served as candidates. Serial 10‐fold dilutions of co‐transformed yeast cells on double‐dropout (DDO) and quadruple‐dropout (QDO) + X‐α‐gal are shown. The experiments were performed twice, with similar results. [file MPP-25-e70002-s008.docx]

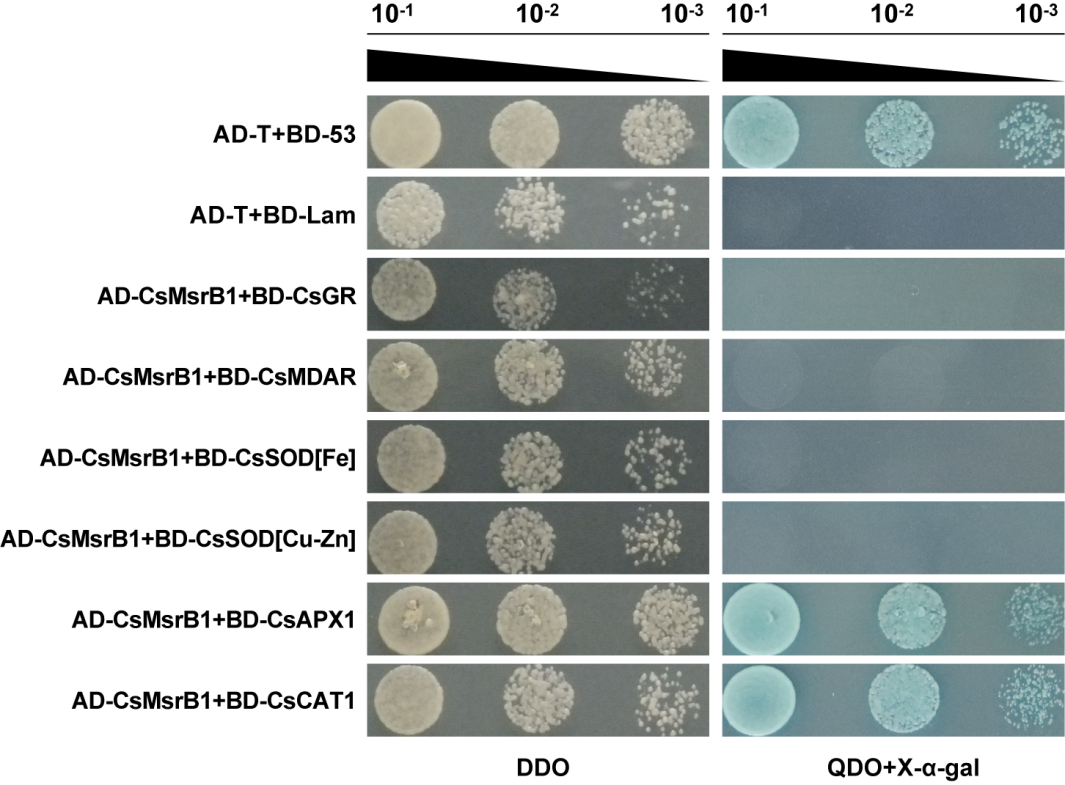
**Figure S5.** Validation of the interactions between CsMsrB1 and enzymatic antioxidants using yeast two-hybrid (Y2H). Glutathione reductase (GR) (XP_006493268.2), monodehydroascorbate reductase (MDAR) (XP_006476500.2), SOD[Fe] (XP_006485042.1), SOD[Cu-Zn] (XP_006471806.2), ascorbate peroxidase 1 (APX1, XP_015388868.1), and catalase 1 (CAT1, XP_015388868.1) served as candidates. Serial 10-fold dilutions of co-transformed yeast cells on DDO and QDO+X-α-gal are shown. The experiments were performed twice, with similar results.
