# Supplementary material for: Effector CLas0185 targets methionine sulphoxide reductase B1 of Citrus sinensis to promote multiplication of ‘Candidatus Liberibacter asiaticus’ via enhancing enzymatic activity of ascorbate peroxidase 1
Source: Mol Plant Pathol. 2024 Aug 31;25(9):e70002. doi: 10.1111/mpp.70002 (PMC11365454; doi:10.1111/mpp.70002)
Supplement: Supplementary file 6 — FIGURE S6. Sequence analysis of ascorbate peroxidases (APXs) and the inter‐relationship between CsMsrB1 and CsAPXs. (a) Phylogenetic analysis of APX homologues within Citrus sinensis (6), Nicotiana tabacum (3), Arabidopsis thaliana (6), and Zea mays (7). Plant species and their corresponding symbols are listed on the right top of the figure. Neighbour‐joining method was applied to generate the phylogeny with 1000 bootstrap replicates. Bootstrap values are indicated at each node. Scale bar: 0.20. According to amino sequence identity, APX homologues were divided into five clusters (I–V). (b) Alignment of amino acid sequences of CsAPXs. Black shading indicates 100% similarity across sequences. (c) The interactions between CsMsrB1 and CsAPXs were verified with pairwise yeast two‐hybrid assays. Serial 10‐fold dilutions of co‐transformed yeast cells on double‐dropout (DDO) and quadruple‐dropout (QDO) + X‐α‐gal are shown. The experiments were performed twice, with similar results. [file MPP-25-e70002-s006.docx]

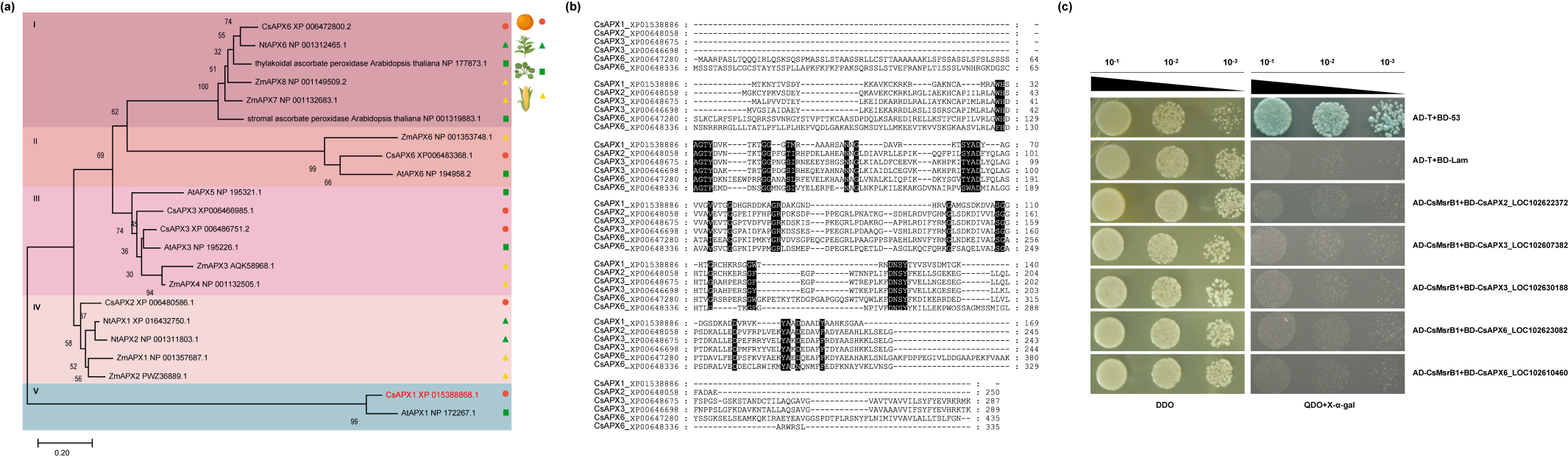


**Figure S6. Sequence analysis of APXs and the interrelationship between CsMsrB1 and CsAPXs.**

1. Phylogenetic analysis of APX homologous within *Citrus sinensis* (6), *Nicotiana* *tobaccum* (3), *Arabidopsis thaliana* (6), and *Zea mays* (7). Plant species and their corresponding symbols are listed on the right top of the figure. Neighbor-Joining method was applied to generate the phylogeny with 1 000 bootstrap replicates. Bootstrap values are indicated at each node. Scale bar: 0.20. According to amino sequence identity, APX homologs were divided into five clusters (I-V). (b) Alignment of amino acid sequences of CsAPXs. Black shading indicates 100% similarity across sequences. (c) The interactions between CsMsrB1 and CsAPXs were verified with pair-wise Y2H assays. Serial 10-fold dilutions of co-transformed yeast cells on DDO and QDO+X-α-gal are shown. The experiments were performed twice, with similar results.
