# Supplementary material for: Effector CLas0185 targets methionine sulphoxide reductase B1 of Citrus sinensis to promote multiplication of ‘Candidatus Liberibacter asiaticus’ via enhancing enzymatic activity of ascorbate peroxidase 1
Source: Mol Plant Pathol. 2024 Aug 31;25(9):e70002. doi: 10.1111/mpp.70002 (PMC11365454; doi:10.1111/mpp.70002)
Supplement: Supplementary file 7 — FIGURE S7. Transient expression and virus‐induced gene silencing of CsAPX1 in Citrus sinensis. (a) Immunoblot analysis. CsAPX1 and GUS C‐terminally fused with HA were expressed in citrus leaves through Agrobacterium infiltration. Protein was extracted at 3 days post‐infiltration, and was verified by immunoblotting with an anti‐HA antibody, and equal loading of each sample is confirmed by immunoblot of RuBisCO. Mock represents the wild‐type (WT) negative control. (b) Reverse transcription‐PCR was used to determine the fragment insertion. CLBV:GUS_1–7 represent CLBV:GUS inoculated citrus plants, and CLBV:CsAPX1_1–5 represent CLBV:CsAPX1 inoculated citrus plants. ‘−’ represents WT as the negative control. CLBV:GUS served as the control for both CLBV:CsMsrB1 and CLBV:CsAPX1, thereby the figure of CLBV:GUS agarose gel electrophoresis is the same as that used in Figure S4b. (c, d) Relative expression levels of CsAPX1 in Wanjincheng. Transcripts levels of CsAPX1 measured with reverse transcription‐quantitative PCR were normalized to levels in GUS‐OE/CLBV:GUS control using the CsGAPDH as endogenous control. The differences were analysed using Student’s t test (**p < 0.01, n = 3). [file MPP-25-e70002-s010.docx]

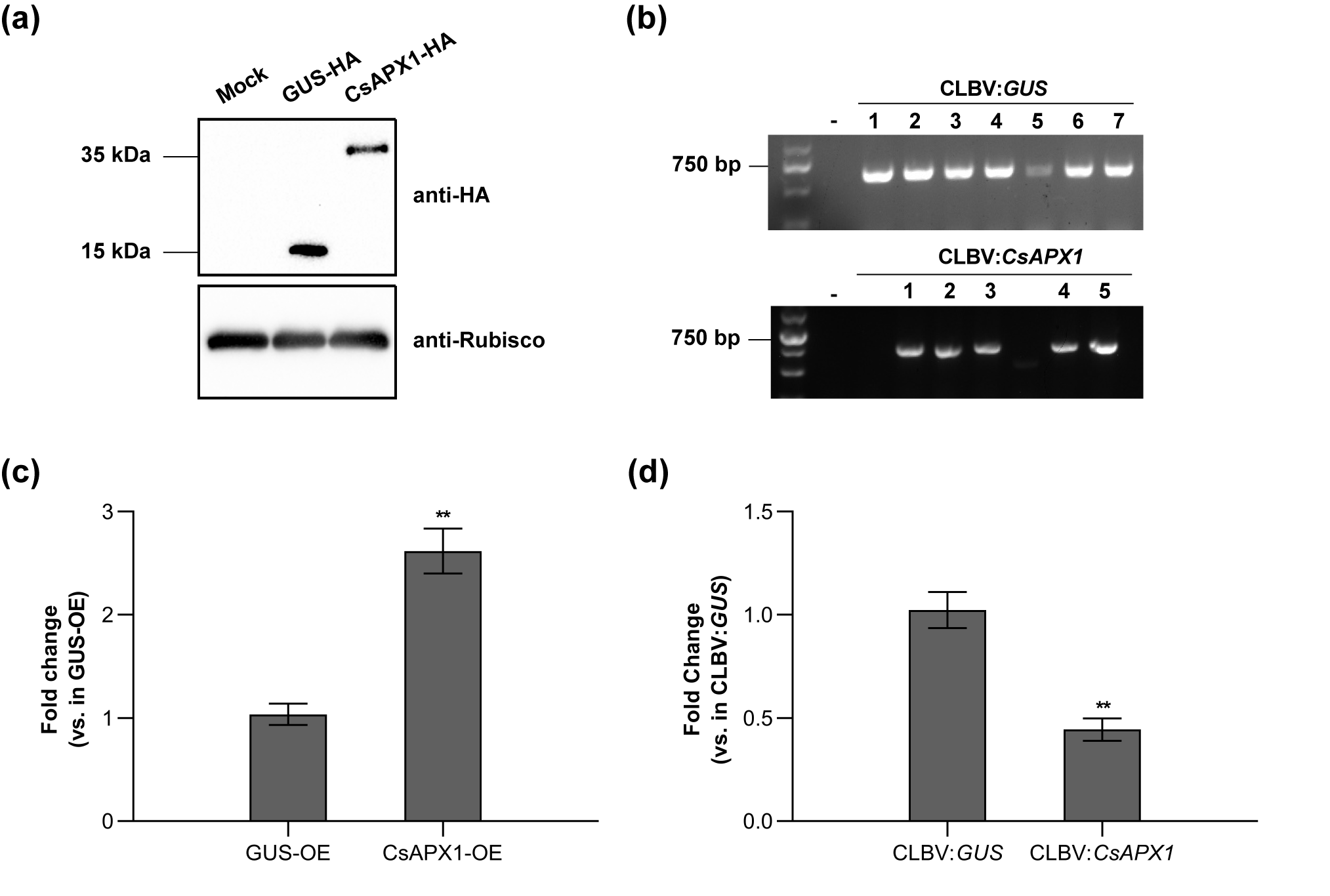
**Figure S7.** Transient expression and VIGS of *CsAPX1* in *C*. *sinensis*. (a) Immunoblot analysis. CsAPX1 and GUS C-terminally fused with HA were expressed in citrus leaves through agrobacterium infiltration. Protein was extracted at 3 dpi, and was verified by immunoblotting with a HA antibody, and equal loading of each sample is confirmed by immunoblot of Rubisco. Mock represents the wild-type negative control. **(b)** RT-PCR was used to determined the fragment insertion. CLBV:*GUS_*1-7 represent CLBV:*GUS* inoculated citrus plants, and CLBV:*CsAPX1*_1-5 represent CLBV:*CsAPX1* inoculated citrus plants. ‘-’ represents WT as the negative control.. CLBV:*GUS* served as the control for both CLBV:*CsMsrB1 and* CLBV:*CsAPX1*, thereby the figure of CLBV:*GUS* agarose gel electrophoresis is the same as that used in Figure S4b. **(c, d)** Relative expression levels of *CsAPX1* in Wanjincheng. Transcripts levels of *CsMsrB1* measured with qRT-PCR were normalized to levels in GUS-OE/CLBV:*GUS* control using the *CsGAPDH* as endogenous control. The differences were analyzed using Student’s *t*-test (***P*<0.01, ****P*<0.001, n=3).
