# Supplementary material for: Effector CLas0185 targets methionine sulphoxide reductase B1 of Citrus sinensis to promote multiplication of ‘Candidatus Liberibacter asiaticus’ via enhancing enzymatic activity of ascorbate peroxidase 1
Source: Mol Plant Pathol. 2024 Aug 31;25(9):e70002. doi: 10.1111/mpp.70002 (PMC11365454; doi:10.1111/mpp.70002)
Supplement: Supplementary file 8 — FIGURE S8. Generation of genetic transformation hairy roots overexpressing/silencing CsAPX1 in huanglongbing‐diseased citrus plants. (a) Generation of transgenic hairy roots overexpressing/silencing CsAPX1. Structures of the pLGN‐CsAPX1 applied for the overexpression assay, and pGN‐CsAPX1‐RNAi for gene silencing. Identification of transgenic plants with PCR and β‐glucuronidase GUS) staining. M, DNA marker; WT, wild‐type control; CsAPX1‐OE#, transgenic lines expressing CsAPX1. (b) Phenotypes of Agrobacterium rhizogenes‐induced hairy root. Scale bar: 10 mm. (c, d) Relative expression levels of CsAPX1 in citrus plants. Transcripts levels of CsAPX1 measured with reverse transcription‐quantitative PCR were normalized to levels in ‘Candidatus Liberibacter asiaticus’‐infected WT using the CsGAPDH as endogenous control. The differences were analysed using Student’s t test (**p < 0.01; ***p < 0.001, n = 4). [file MPP-25-e70002-s001.docx]

**
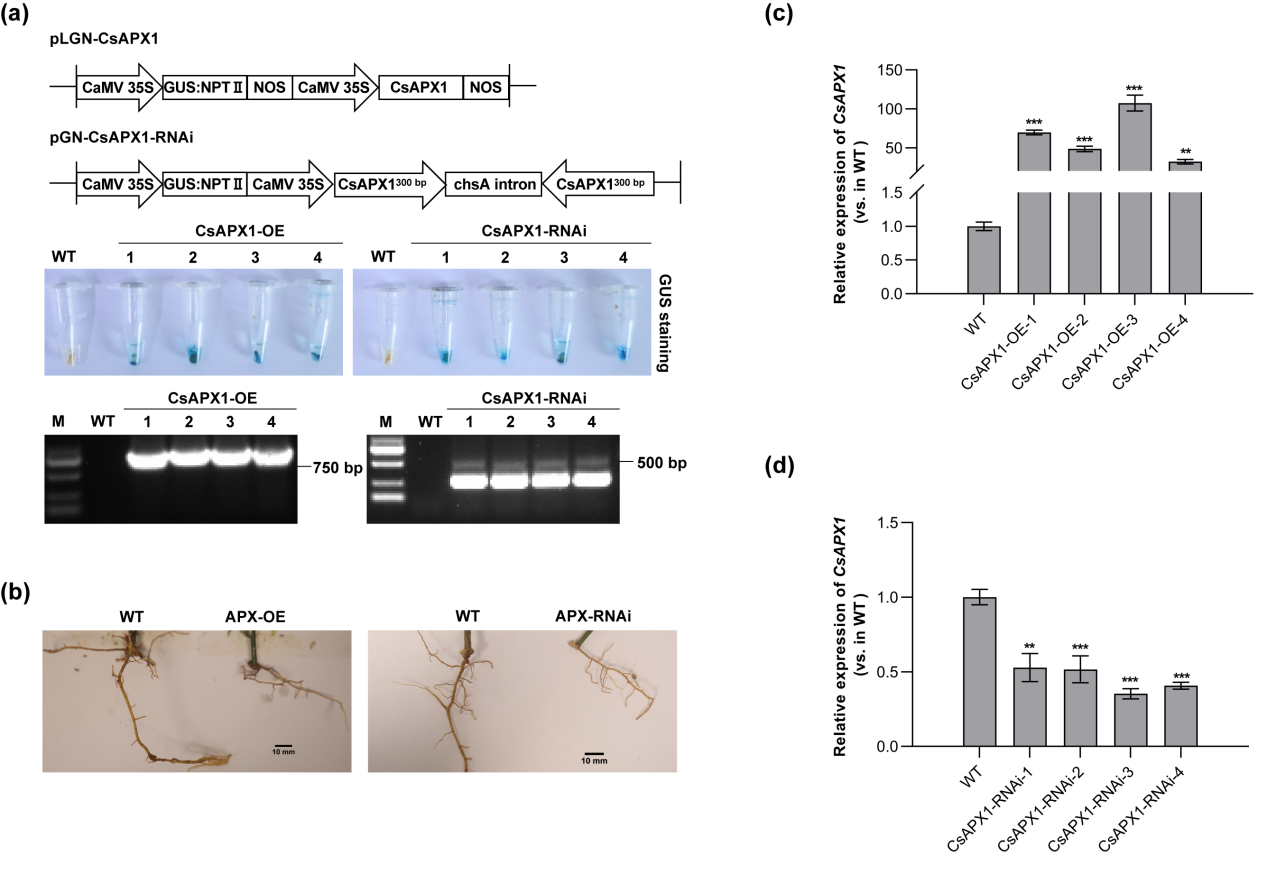
Figure S8.** Generation of genetic transformation hairy roots overexpressing/silencing *CsAPX1* in HLB-diseased citrus plants. **(a)** Generation of transgenic hairy roots overexpressing/silencing *CsAPX1*. Structures of the pLGN-CsAPX1 applied for the overexpression assay, and pGN-CsAPX1 for gene silencing. Identification of transgenic plants with PCR and GUS staining. M, DNA marker; WT, wild-type control; CsAPX1-OE#, transgenic lines expressing *CsAPX1*. **(b)** Phenotypes of *Agrobacterium* *rhizogenes-*induced hairy root. Scale bar: 10 mm. **(c and d)** Relative expression levels of *CsAPX1* in citrus plants. Transcripts levels of *CsAPX1* measured with qRT-PCR were normalized to levels in *C*Las-infected WT using the *CsGAPDH* as endogenous control. The differences were analyzed using Student’s *t*-test (***p*<0.01, ****p*<0.001, n=4).
