# Supplementary material for: Effector CLas0185 targets methionine sulphoxide reductase B1 of Citrus sinensis to promote multiplication of ‘Candidatus Liberibacter asiaticus’ via enhancing enzymatic activity of ascorbate peroxidase 1
Source: Mol Plant Pathol. 2024 Aug 31;25(9):e70002. doi: 10.1111/mpp.70002 (PMC11365454; doi:10.1111/mpp.70002)
Supplement: Supplementary file 9 — TABLE S1. Candidate proteins that interact with CLas0185 via yeast two‐hybrid assay. [file MPP-25-e70002-s005.docx]

**Table S1** Candidate proteins that interact with *C*Las0185 via Y2H assay

| Number | Gene ID in NCBI database | Length (aa) | Annotation |
| --- | --- | --- | --- |
| 1 | LOC102627665 | 380 | disulfide-isomerase like 2-1 |
| 2 | LOC102624107 | 313 | shikimate kinase 1 |
| 3 | LOC102628757 | 329 | uric acid degradation bifunctional protein TTL |
| 4 | LOC102610947 | 180 | constitutive photomorphogenesis protein 10 |
| 5 | LOC102606697 | 198 | rac-like GTP-binding protein RHO1 |
| 6 | LOC102616514 | 507 | serine carboxypeptidase-like |
| 7 | LOC102618702 | 313 | 1-acyl-sn-glycerol-3-phosphate acyltransferase |
| 8 | LOC102611194 | 198 | axial regulator YABBY 5 |
| 9 | LOC102620912 | 202 | peptide methionine sulfoxide reductase B1 |
| 10 | LOC102578015 | 342 | glyceraldehyde-3-phosphate dehydrogenase GAPC1 |
| 11 | LOC102614495 | 606 | glyceraldehyde-3-phosphate dehydrogenase A |
| 12 | LOC102622895 | 203 | ribonuclease 3-like protein 1 |
| 13 | LOC102625183 | 122 | 60S ribosomal protein L31 |
| 14 | LOC102606640 | 103 | cold and drought-regulated protein CORA-like |
| 15 | LOC102618220 | 511 | ninja-family protein mc410 |
| 16 | LOC102617233 | 1101 | protein HIRA |
| 17 | LOC102615191 | 102 | small ubiquitin-related modifier 1-like |
| 18 | LOC102611533 | 324 | clathrin light chain 1-like |
| 19 | LOC102610420 | 259 | clathrin light chain 2 |
| 20 | LOC102615388 | 540 | la-related protein 6B |
| 21 | LOC102630752 | 353 | histone deacetylase 2 |
| 22 | LOC102610914 | 366 | zinc finger protein CONSTANS-LIKE 2 |
| 23 | LOC102627054 | 300 | peroxisomal 2,4-dienoyl-CoA reductase |
| 24 | LOC102607595 | 221 | tetraspanin-19 |
| 25 | LOC102609385 | 655 | DEK domain-containing chromatin-associated protein 4 |
| 26 | LOC102619242 | 853 | heat shock 70 kDa protein 15 |
| 27 | LOC102622657 | 327 | high mobility group B protein 10 |
| 28 | LOC102614932 | 304 | adenylate kinase |
| 29 | LOC102615090 | 435 | zinc finger CCCH domain-containing protein 48 |
| 30 | LOC102620954 | 631 | factor of DNA methylation 4 |
| 31 | LOC102629555 | 220 | deoxyuridine 5'-triphosphate nucleotidohydrolase |
| 32 | LOC102622151 | 195 | deoxyuridine 5'-triphosphate nucleotidohydrolase-like |
| 33 | LOC102621376 | 123 | 40S ribosomal protein S20-2 |
| 34 | LOC102621342 | 1889 | protein REDUCED CHLOROPLAST COVERAGE 1 |
| 35 | LOC102619774 | 279 | SNF1-related protein kinase regulatory subunit beta-1 |
| 36 | LOC102622031 | 201 | dehydration-responsive element-binding protein 1B-like |
| 37 | LOC102611474 | 373 | STRICTOSIDINE SYNTHASE-LIKE 4-like |
| 38 | LOC102627743 | 220 | peptidyl-prolyl cis-trans isomerase FKBP18 |
| 39 | LOC102623286 | 832 | BEL1-like homeodomain protein 4 |
| 40 | LOC102609089 | 1621 | ferredoxin-dependent glutamate synthase 1 |
| 41 | LOC102610142 | 606 | MACPF domain-containing protein |
| 42 | LOC102616023 | 339 | SODIUM POTASSIUM ROOT DEFECTIVE 3 |
| 43 | LOC102627510 | 146 | eukaryotic translation initiation factor 1A |
| 44 | LOC102612882 | 256 | agamous-like MADS-box protein AGL15 |
| 45 | LOC102619648 | 286 | plastid-lipid-associated protein 6 |
| 46 | LOC102624603 | 778 | elongation factor G-2 |
| 47 | LOC102614429 | 133 | small nuclear ribonucleoprotein SmD3b |
| 48 | LOC102609127 | 780 | subtilisin-like protease SBT1.6 |
| 49 | LOC102623505 | 375 | protein SGT1 homolog B |
