# Supplementary material for: Effector CLas0185 targets methionine sulphoxide reductase B1 of Citrus sinensis to promote multiplication of ‘Candidatus Liberibacter asiaticus’ via enhancing enzymatic activity of ascorbate peroxidase 1
Source: Mol Plant Pathol. 2024 Aug 31;25(9):e70002. doi: 10.1111/mpp.70002 (PMC11365454; doi:10.1111/mpp.70002)
Supplement: Supplementary file 10 — TABLE S2. Strains and plasmids used in this study. [file MPP-25-e70002-s004.docx]

**Table S2** Strains and plasmids used in this study

| **Strains and vectors** | **Characteristics** | **Source** |
| --- | --- | --- |
| **Strains** |  |  |
| DH5α | *Escherichia coli strain* for plasmid construction | Shanghai Weidi Biotechnology Co.，Ltd |
| Rosetta-gami 2 (DE3) | Prokaryotic expression strain |  |
| GV3101(PJIC SA_Rep) | *Agrobacterium tumefaciens* stain |  |
| EHA105 |  |  |
| GV3101 |  |  |
| K599 | *A*. *rhizogenes* stain |  |
| Y2H Gold | *Saccharomyces cerevisiae* |  |
| **Vectors** |  |  |
| PVX | *Nicotiana benthamiana* transient expression | Lab collection |
| AD/BD | Vectors for yeast two-hybrid assays |  |
| pGEX-6P-1 | GST tag protein expression vector |  |
| pET-28a | His tag protein expression vector |  |
| pCLBV | Vector for virus induced gene silencing |  |
| pLGN | Vector for overexpression | from Prof. Xiuping Zou |
| pGN | Vector for RNA interference |  |
| nLuc/cLuc | Vectors for luciferase imaging assays | from Prof. Jian-Min Zhou |
