# Supplementary material for: Effector CLas0185 targets methionine sulphoxide reductase B1 of Citrus sinensis to promote multiplication of ‘Candidatus Liberibacter asiaticus’ via enhancing enzymatic activity of ascorbate peroxidase 1
Source: Mol Plant Pathol. 2024 Aug 31;25(9):e70002. doi: 10.1111/mpp.70002 (PMC11365454; doi:10.1111/mpp.70002)
Supplement: Supplementary file 11 — TABLE S3. Primers used in this study. [file MPP-25-e70002-s002.docx]

**Table S3** Primers used in this study

| **Name** |  | **Primer sequence** （**5'-3'**） | **Restriction site** |
| --- | --- | --- | --- |
| **Transient expression in *Nicotiana benthamiana*** | | |  |
| *C*Las0185 | F | ATGTGTGATTCGGAATCGAAAAA |  |
|  | R | CAGATCATCCTGAATCACCAAGG |  |
| GUS | F | CCTTGGTGATTCAGGATGATCTG |  |
|  | R | GATGCGCTGCGAATCGGGA |  |
| CLas0185-GUS | F | ATGTGTGATTCGGAATCGAAAAA |  |
|  | R | GATGCGCTGCGAATCGGGA |  |
| PVX-*C*Las0185 | F | tcagcaccagctagcatcgatATGTGTGATTCGGAATCGAAAAA | *Cla*Ӏ/*Sal*Ӏ |
|  | R | tcaagcttatcggcggtcgacTTAAATCACCAAGGTCACGTCG |  |
| PVX-*C*Las0185-GUS-HA | F | tcagcaccagctagcatcgatATGTGTGATTCGGAATCGAAAAA | *Cla*Ӏ |
|  | R | aacatcgtatgggtaatcgatGATGCGCTGCGAATCGGG |  |
| PVX-BAX-Flag | F | tcagcaccagctagcatcgatATGGACGGGTCCGG |  |
|  | R | gtcatccttgtaatcatcgatCGCGGCCGCG |  |
| **Yeast two-hybrid assay** | | |  |
| AD-CsMsrB1 | F | gccatggaggccagtgaattcATGATAATGGCGATTTCGCATA | *Eco*RӀ/*Bam*HӀ |
|  | R | cagctcgagctcgatggatccTTAAATCACCAAGGTCACGTCG |  |
| BD-*C*Las0185 | F | gccatggaggccagtgaattcATGTGTGATTCGGAATCGAAAAA | *Eco*RӀ/*Sal*Ӏ |
|  | R | atgcggccgctgcaggtcgacTTAAATCACCAAGGTCACGTCG |  |
| BD-CsGR | F | gccatggaggccagtgaattcATGGCAAGGAAGATGCTTA |  |
|  | R | atgcggccgctgcaggtcgacTTACAGATTTGTCTTTGGATTGC |  |
| BD-CsMDAR | F | gccatggaggccagtgaattcATGTCTACAGCTCGAAAA |  |
|  | R | atgcggccgctgcaggtcgacCTAAACTGCAGCCTC |  |
| BD-CsSOD[Fe] | F | gccatggaggccagtgaattcATGGCTGCAGCAGCTG |  |
|  | R | atgcggccgctgcaggtcgacTCACTCGGATTCTGACTCATCAC |  |
| BD-CsSOD[Cu-Zn] | F | gccatggaggccagtgaattcATGGTGAAAGCAGTTGCA |  |
|  | R | atgcggccgctgcaggtcgacTCACCCTTGGAGGCCA |  |
| BD-CsAPX1 | F | gccatggaggccagtgaattcATGACGAAGAATTACCCCACTGTT |  |
|  | R | atgcggccgctgcaggtcgacTTATCCTCCTCCGGCTTC |  |
| BD-CsCAT1 | F | gccatggaggccagtgaattcATGGATCCCTACAAATTGCTT |  |
|  | R | atgcggccgctgcaggtcgacTCAAATGCTTGGCCTCAC |  |
| BD-CsAPX2 | F | gccatggaggccgaattcATGGGAAAGTGTTATCCAAAA |  |
|  | R | atgcggccgctgcaggtcgacTTACTCAGCATCCGCAAATCC |  |
| BD-CsAPX3_LOC102607382 | F | gccatggaggccgaattcATGGCTTTACCGGTCGTTGAC |  |
|  | R | atgcggccgctgcaggtcgacTCACTTCATCCTTTTGCGAAC |  |
| BD-CsAPX3_LOC102630118 | F | gccatggaggccgaattcATGGTGGGAAGCATCGCAATT |  |
|  | R | atgcggccgctgcaggtcgacTTACTTGGTTTTTCTGTGAAC |  |
| BD-CsAPX6_LOC102623082 | F | gccatggaggccgaattcATGAGTTCATCAACTGCCT |  |
|  | R | atgcggccgctgcaggtcgacTCACAAGCTTCTCCACCT |  |
| **Luciferase imaging assay** | | |  |
| *C*Las0185-nLuc | F | acgggggacgagctcggtaccATGTGTGATTCGGAATCGAAAAA | *Bam*HӀ/*Sal*Ӏ |
|  | R | aacatcgtatgggtagtcgacAATCACCAAGGTCACGTCGATAA |  |
| CsMsrB1-cLuc | F | acgggggacgagctcggtaccATGATAATGGCGATTTCGCATA |  |
|  | R | cgcgtacgagatctggtcgacTTTTGGCTTCAGTTTTAAGGAAGC |  |
| CsAPX1-nLuc | F | acgggggacgagctcggtaccATGACGAAGAATTACCCCACTGTT |  |
|  | R | aacatcgtatgggtagtcgacTCCTCCTCCGGCTTC |  |
| HA-nLuc | F | acgggggacgagctcggtaccATGTACCCATACGATGTTCCT |  |
|  | R | aacatcgtatgggtagtcgacAGCGTAATCTGGAACGTC |  |
| HA-cLuc | F | acgggggacgagctcggtaccATGTACCCATACGATGTTCCT |  |
|  | R | cgcgtacgagatctggtcgacAGCGTAATCTGGAACGTC |  |
| **Pull-down assay** | | |  |
| GST*-C*Las0185 | F | ttccaggggcccctgggatccATGTGTGATTCGGAATCGAAAAA | *Bam*HӀ/*Sal*Ӏ |
|  | R | gatgcggccgctcgagtcgacTTAAATCACCAAGGTCACGTCG |  |
| GST-CsAPX | F | ttccaggggcccctgggatccATGACGAAGAATTACCCCACTGTT |  |
|  | R | gatgcggccgctcgagtcgacTTATCCTCCTCCGGCTTC |  |
| His-CsMsrB1 | F | cagcaaatgggtcgcggatccATGATAATGGCGATTTCGCATA | *Bam*HӀ/*Eco*RӀ |
|  | R | ttgtcgacggagctcgaattcTTAAATCACCAAGGTCACGTCG |  |
| **Transient expression in *Citrus sinensis*** | | |  |
| pLGN-CsAPX1-HA | F | cggggatccactagtgtcgacATGACGAAGAATTACCCCACTGTT | *Sal*Ӏ |
|  | R | aacatcgtatgggtagtcgacTCCTCCTCCGGCTTC |  |
| pLGN-GUS-HA | F | cggggatccactagtgtcgacatgCCTTGGTGATTcaggatgatctg |  |
|  | R | aacatcgtatgggtagtcgacGATGCGCTGCGAATCGGG |  |
| pLGN-CsMsrB1-Flag | F | cggggatccactagtgtcgacATGATAATGGCGATTTCGCATA |  |
|  | R | gtcatccttgtaatcgtcgacTTTTGGCTTCAGTTTTAAGGAAGC |  |
| pLGN-GUS-Flag | F | cggggatccactagtgtcgacatgCCTTGGTGATTcaggatgatctg |  |
|  | R | gtcatccttgtaatcgtcgac gatgcgctgcgaatcggga |  |
| **Generation of transgenic *C*. *sinensis*** | | |  |
| pLGN-*C*Las0185 | F | cggggatccactagtgtcgacATGTGTGATTCGGAATCGAAAAA | *Sal*Ӏ/*EcoR*Ӏ |
|  | R | tctcattaaagcagggaattcTTAAATCACCAAGGTCACGTCG |  |
| pLGN-CsAPX1 | F | cggggatccactagtgtcgacATGACGAAGAATTACCCCACTGTT |  |
|  | R | tctcattaaagcagggaattcTTATCCTCCTCCGGCTTC |  |
| pLGN-CsMsrB1 | F | cggggatccactagtgtcgacATGATAATGGCGATTTCGCATA |  |
|  | R | tctcattaaagcagggaattcTTAAATCACCAAGGTCACGTCG |  |
| pGN-CsAPX1-RNAi | F1 | gatgatatcccatggggcgcgccATGAGCGACAAGGATATTGTTGC | *Asc*I/*Swa*I |
|  | R1 | aagaaattcttacacatttaaatGGCTTCAGCAAATCCTAGCTCA |  |
|  | F2 | agggaattcctgcaggtcgacATGAGCGACAAGGATATTGTTGC | *Bam*HӀ/*Sal*Ӏ |
|  | R2 | aatttgcaggtatttggatccGGCTTCAGCAAATCCTAGCTCA |  |
| pGN-CsMsrB1-RNAi | F1 | gatgatatcccatggggcgcgccATGTTTACGGGGGAATATTGGA | *Asc*I/*Swa*I |
|  | R1 | aagaaattcttacacatttaaatTTTTGGCTTCAGTTTTAAGGAAGC |  |
|  | F2 | agggaattcctgcaggtcgacATGTTTACGGGGGAATATTGGA | *Bam*HӀ/*Sal*Ӏ |
|  | R2 | aatttgcaggtatttggatccTTTTGGCTTCAGTTTTAAGGAAGC |  |
| **Virus-induced gene silencing** | | |  |
| CLBV:*GUS* | F | ctcttagaaatgtagcccgggcaggatgatctggacgaa | *Sma*I |
|  | R | tgccagaattcgggacccgggaagatctaccatgtacagctcg |  |
| CLBV:*CsAPX* | F | ctcttagaaatgtagcccgggccagcaagctgataaaggtca |  |
|  | R | tgccagaattcgggacccggggaattaccccactgttagcga |  |
| CLBV:*CsMsrB1* | F | ctcttagaaatgtagcccgggttttggcttcagttttaagga |  |
|  | R | tgccagaattcgggacccgggccattgcatttgttgtgacac |  |
| **qRT-PCR** | | |  |
| *qCLas0185* | F | ATGGATCATAGAAAGAAA |  |
|  | R | TTAAATCACCAAGGTCAC |  |
| *CLasgyrA* | F | GTATGGCACAGGACTGGTCT |  |
|  | R | GTTAGGGCGGAAATCAACAGT |  |
| *18S rRNA* | F | AATTGTTGGTCTTCAACGAGGAA |  |
|  | R | AAAGGGCAGGGACGTAGTCAA |  |
| *CsGAPDH* | F | CATCCCTCAGCACCTTCC |  |
|  | R | CCAACCTTAGCACTTCTCC |  |
| *qCsPR1* | F | AAATGTGGGTGAATGAGAAAGC |  |
|  | R | ATTATTGTTGCACGTCACCTTG |  |
| *qCsPR2* | F | TTCCACTGCCATCGAAACTG |  |
|  | R | GTAATCTTGTTTAAATGAGCCTCTTG |  |
| *qCsPR5* | F | CACCATTGCCAATAACCCTAATG |  |
|  | R | GGGACAGTTACCGTTAAGATCAG |  |
| *qCsPP2* | F | GTTGGTTTCATGGGCGTTCC |  |
|  | R | TGAAGCTCTTTCTACCGCCG |  |
| *qCsMsrB1* | F | atgataatggcgatttcgcat |  |
|  | R | acttatagaagcataatcaac |  |
| *qCsAPX1* | F | CTGAGAAGAACTGCGCTCCA |  |
|  | R | CTTGAACGGCTCCAAAAGCC |  |
